# Lnk deficiency attenuates the immunosuppressive capacity of MDSCs via ferroptosis to suppress tumor development

**DOI:** 10.1038/s41419-025-07948-8

**Published:** 2025-08-12

**Authors:** Jingwen Zhou, Hui Yin, JiaHua Pan, Rui Yin, Xin Wei, Min Shen, Liangliang Cai, Ziqi Liu, Jie Zhao, Wenyan Chen, Ruoxun Wang, Xinrui Lan, Wenshu Han, Dongkun Li, Xiaoyu Zhu, Weijuan Gong, Li Qian

**Affiliations:** 1 Key Laboratory of the Jiangsu Higher Education Institutions for Nucleic Acid & Cell Fate Regulation (Yangzhou University), School of Medicine, Yangzhou University, Yangzhou, 225001 China; 2 Department of Clinical Laboratory Medicine, the Air Force Hospital of Eastern Theater Command, PLA, Nanjing, 210001 China; 3 Department of Respiratory Medicine, Taizhou Second People’s Hospital Affiliated to Yangzhou University, Yangzhou University, Yangzhou, 225001 China; 4 Department of Ophthalmology, the Affiliated Hospital of Yangzhou University, Yangzhou, 225001 China

**Keywords:** Immunology, Cell biology

## Abstract

A subset of immature myeloid cells known as myeloid-derived suppressor cells (MDSCs) play an immunosuppressive role and actively stimulate the growth of tumors. Lymphocyte adaptor protein (Lnk) regulates the development of hematopoietic stem cells and inflammatory CD8^+^ T cells by inhibiting cytokine signaling. However, it is unclear how Lnk regulates the function of MDSCs during tumorigenesis. Here, using *Lnk*^*–/–*^ mice, we showed that Lnk deficiency inhibited tumor growth in an MDSC-dependent manner. Mechanistically, we demonstrated that Lnk deficiency weakened the immunosuppressive effects of MDSCs through ferroptosis. In addition, Lnk deficiency-induced ferroptosis was regulated by the Flt3/STAT1/IRF1/Alox12 axis. Besides, Lnk was more highly expressed in MDSCs from lung cancer patients. Knocking down Lnk in human MDSCs resulted in increased TNF-α and decreased Arg-1 expression. These findings demonstrate that the role of Lnk is vital in the immunosuppressive ability of MDSCs and offers a novel target for cancer treatment.

## Introduction

Tumor progression is intimately linked to the development and activity of immune cells, such as myeloid-derived suppressor cells (MDSCs, CD11b^+^Gr-1^+^) [1–3]. MDSCs not only directly promote tumor growth by secreting growth-promoting mediators but also inhibit T-cell function [4, 5], thereby facilitating tumor immune escape [6, 7]. MDSCs suppress T cell-response by expressing arginase-1 (Arg-1) and inducible nitric oxide synthase (iNOS) [8, 9]. Additionally, they also prevent T cells activation through expressing reactive oxygen species (ROS) and programmed death-ligand 1 (PD-L1) [10]. Because of their powerful immunosuppressive activity, MDSCs are a major obstacle for cancer immunotherapy [11].

Lymphocyte adaptor protein (Lnk) is a member of the SH2B family and is a negative regulator of cytokine signaling [12, 13]. Lnk is involved in negatively regulating multiple signal transduction pathways through SH2 domains and tyrosine phosphorylation sites, for example, the signal transducer and activator of transcription (STAT), Janus kinase (Jak), and phosphatidylinositol 3 kinase pathways, among others [14–16]. In addition, Lnk can bind directly to receptors such as Fms-related tyrosine kinase 3 (Flt3) and platelet-derived growth factor receptor, thereby inhibiting downstream signaling [17–19]. Lnk is mainly expressed in hematopoietic cells and negatively regulates the growth of hematopoietic stem cells, thus playing a role in malignant diseases of the hematopoietic system, such as myelodysplastic syndrome and acute lymphoblastic leukemia [20]. Lnk has also been reported to control the activity of immune cells; for example, Lnk inhibits the proliferation of inflammatory CD8^+^ T cells to maintain intestinal homeostasis [21]. Lnk also modulates the ability of dendritic cells (DCs) to inhibit the differentiation of regulatory T cells (Tregs) [22, 23]. However, whether Lnk is expressed in MDSCs and involved in regulating the function of MDSCs, thereby indirectly controlling tumor progression, remains unclear.

Ferroptosis is an iron-mediated form of regulated cell death that caused by the unrestricted accumulation of lipid peroxidation, glutathione (GSH) inhibition and iron overload, which subsequently damage the cell membranes [24]. When antioxidant defenses are weakened, phospholipids (PLs) embedded in membranes experience excessive peroxidation, resulting in cell disruption. Glutathione peroxidase 4 (GPX4) is a PL-hydroperoxidase against ferroptosis that uses GSH as a cofactor to catalyze the reduction of oxidized lipids. GSH is a major cellular antioxidant, and its excessive consumption leads to a loss of intracellular redox homeostasis [25, 26]. In addition, the overactivation of Lipoxygenases (LOXs), especially Alox12, can convert polyunsaturated fatty acids (PUFAs) into lipid peroxides, which directly drive ferroptosis [27, 28]. Ferroptosis has been extensively studied in the treatment of multiple cancers. In cancer research, ferroptosis has drawn increasing attention to immune cells as well as tumor cells [29]. Recently, a single-cell transcriptome analysis of tumor-infiltrating MDSCs from human colorectal cancer showed enrichment of ferroptosis pathway genes [30]. In addition, there is evidence that inducing MDSCs ferroptosis can reduce the proportion of MDSCs in tumor and spleen to suppress tumor growth [31]. These studies suggest that ferroptosis may lead to a decrease in the number of MDSCs, but the specific effects of ferroptosis on the function of MDSCs and the regulatory mechanisms are not fully understood.

At present, many regulatory factors of ferroptosis have been found. Ferritin and transferrin/transferrin receptors are key to protecting cells from ferroptosis [32]. Several enzymes and proteins in lipid metabolism and redox systems have been found to promote ferroptosis, such as acyl-CoA synthetase long-chain family member 4 (ACSL4) and lactate transporter 3 (LPCAT3), and serve as ferroptosis-related markers [24, 33]. Transcription factors play an important role in ferroptosis, for example P53 can induce ferroptosis [25]. However, the regulatory role of Lnk in ferroptosis is still unclear.

In our present study, we found that Lnk loss restrained tumor progression in mice in an MDSC-dependent manner. Moreover, Lnk deficiency impaired the immune-inhibitory activity of MDSCs through ferroptosis. Mechanistically, the expression of Alox12 was mediated by the Flt3/STAT1/IRF1 pathway to initiate ferroptosis induced by Lnk deletion. In addition, knocking down Lnk in human MDSCs resulted in increased TNF-α and decreased Arg-1 expression. Our findings provide new evidence that highlights the critical role of Lnk in tumor progression.

## Material and methods

### Patients

The Ethics Committee of Yangzhou University School of Medicine gave its approval to the project. Blood samples were collected from both healthy individuals and lung cancer patients at Northern Jiangsu People’s Hospital, which is affiliated with Yangzhou University.

### Mouse experiments

All experiments were conducted using eight-week-old male mice. C57BL/6 J wild-type (WT) mice were from the Comparative Medicine Center of Yangzhou University. Lnk-deficient (*Lnk*^*–/*–^) mice on a C57BL/6 background were the gift from Professor Duonan Yu (Yangzhou University, China). The animals were grouped randomly. There is no statistical method to pre-determine the sample size for mice experiment, and this is based on preliminary experimental results. The sample size of each experiment is shown in the legend. The investigator was not blinded to the group allocation of the animals during the experiment. No samples or animals were excluded from the analysis. All animal experiments were conducted in accordance with the approval of the Animal Care and Use Committee of Yangzhou University.

3LL (murine lung cancer) and B16F10 (melanoma) cells were purchased from Wuhan Servicebio Technology Company Limited. A total of 2 × 10^6^ 3LL cells or B16F10 cells in PBS were subcutaneously injected into the right backs of mice.

### Flow cytometry

Dead cells were removed from the analysis by using the Fixable Viability Dye eFluor780. All the antibodies used are shown in Table S1. Using a BD FACSVerse, flow cytometry analysis was carried out, and FlowJo 10 software was used to analyze the data.

### Cell isolation

Splenic MDSCs were sorted from the spleens of tumor-bearing mice using anti-mouse CD11b magnetic particles (BD Bioscience, USA). In some experiments, Splenic MDSCs were sorted by FACSAria. Tumor MDSCs from single-cell suspension samples of tumor tissues were sorted using a BD FACSAria SORP. The purity of MDSCs (CD11b^+^Gr-1^+^) was typically >90%. PMN-MDSCs and M-MDSCs were sorted using a BD FACSAria SORP. Splenic T cells were enriched with CD4 or CD8 magnetic beads (Miltenyi Biotech, Germany).

### Bone marrow (BM)-derived MDSCs

BM cells were isolated from the femurs and tibiae of mice. BM-derived MDSCs were induced in RPMI 1640 medium supplemented with 40 ng/mL GM-CSF (ProSpec, Israel) for 48 h or 72 h.

### RNA sequencing (RNA-seq)

Total RNA of MDSCs was extracted using TRIzol (Tiangen, China) and then used for sequencing by preparing libraries with the NEBNext Ultra II RNA Library Prep Kit and sequencing on the Illumina NovaSeq 6000 platform. Gene expression differences were analyzed using DESeq (v1.30.0) with the criteria of a fold change ≥ 1.5 and p < 0.05 and the R language Pheatmap (v1.0.8) software package.

### In vivo and in vitro functional assays of MDSCs

In experiments testing the function of MDSCs in mice in a laboratory setting, CD4^+^ or CD8^+^ T cells that were activated using anti-mouse CD3/CD28 were cocultured with MDSCs at a ratio of 2:1. In some experiments, 500 μM SMT or 5 μM nor-NOHA was added to the culture medium. IFN-γ levels in T cells and their proliferation were measured using flow cytometry 48 h later. To deplete MDSCs, WT and *Lnk*^–/–^ mice were injected with 3LL cells on day 0 and then given intraperitoneal injections of 200 µg of anti-Gr-1 antibody (BioXcell, China) or an isotype control antibody every 3 days. For in vivo MDSCs adoptive transfer experiments, mice with 3LL tumors were given intravenous injections of either WT or *Lnk*^*–/–*^ MDSCs (2 × 10^6^) or PBS on days 3 and 6 after the tumor cells were injected.

### Arginase and NOS enzymatic activity

Arginase activity in MDSCs was assayed with an arginase assay kit (BioAssay Systems, USA). NOS activity was determined by a nitric oxide synthase assay kit (Beyotime, China).

### Intracellular iron assays

Intracellular Fe^2+^ levels were assessed by flow cytometry via the probe FerroOrange (Dojindo, China). MDSCs were treated with or without Fer-1 for 24 h, stained with 1 μM FerroOrange in the dark at 37 °C for 30 min and assessed by flow cytometry.

### Real-time PCR

Cells were treated with TRIzol reagent to obtain total RNA. cDNA was synthesized by utilizing primers and a reverse transcription kit (Tiangen, China). Next, real-time PCR was performed on a Thermo Real Time PCR system. The relative gene expression levels were determined by the ΔΔCt method using actin as a reference gene. The primers were as follows: 5′- TCCCTCAACCTAGTGCGTTTG -3′ and 5′-CCTCGGGAACGTCGAAGTC-3′ (Alox12); and 5′- TGGTTGGAAGAGGCAAGAAC -3′ and 5′-GTAACAAGGATGGCGAGGAG-3′ (Alox15).

### Lentivirus

shRNA-expressing lentiviral plasmids were cotransfected with a lentivirus packaging system (PAX2 and PMD2) into 293 T cells purchased from Wuhan Servicebio Technology Company Limited. Virus-containing medium were collected 72 h later and filtered through 0.22-μm pore sizes. For viral transduction, the collected lentiviral vector and the infection-promoting agent polybrene were added to the MDSCs for 48 h, after which the infected cells were cultured in fresh media for 24 h. Lentiviruses carrying shRNA were produced using the pLKO.1-puro plasmid. A minimum of two target sequences with the least off-target effects compared with those of noncoding hairpins were selected for the IRF1 gene (IRF1: TRCN0000219079, TRCN0000233968;). The following target sequences were used for the knockdown of human Lnk: 5′-GGGCCATAGACAATCAGTACA-3′ and 5′-TGTACTGATTGTCTA TGGCCC-3′.

### Coimmunoprecipitation (Co-IP)

Cell lysates were incubated with anti-Lnk or anti-Flt3 antibodies or IgG at 4 °C overnight, and the target protein was then pulled down with protein-A/G magnetic beads (MCE, China). The agarose-bound immunoprecipitated complexes were collected after centrifugation, washed and assessed by Western blotting. Original western blots are provided in the Supplemental Material.

### Detection of lipid peroxidation

MDSCs were stained with 5 μM BODIPY 581/591 C11 (Invitrogen, USA). After washing, the cells were suspended in PBS and analyzed using a BD FACS Verse flow cytometer to assess lipid peroxidation.

### MDA and GSH assays

A reduced glutathione assay kit (Beyotime, China) and malondialdehyde test kit (Beyotime, China) were used to quantify the GSH and MDA content in the cells.

### Statistical analyses

The statistical analysis was performed with GraphPad Prism 8. Each assay was repeated a minimum of three times, and the results are presented as the mean values with standard error of the mean (SEM). Statistical significance was assessed using the Mann‒Whitney test and denoted as follows: *, P < 0.05; **, P < 0.01; ***, P < 0.001; “ns”, no significant difference.

## Results

### Lnk deficiency reduced tumor progression in mice in an MDSC-dependent manner

**Fig. 1:**
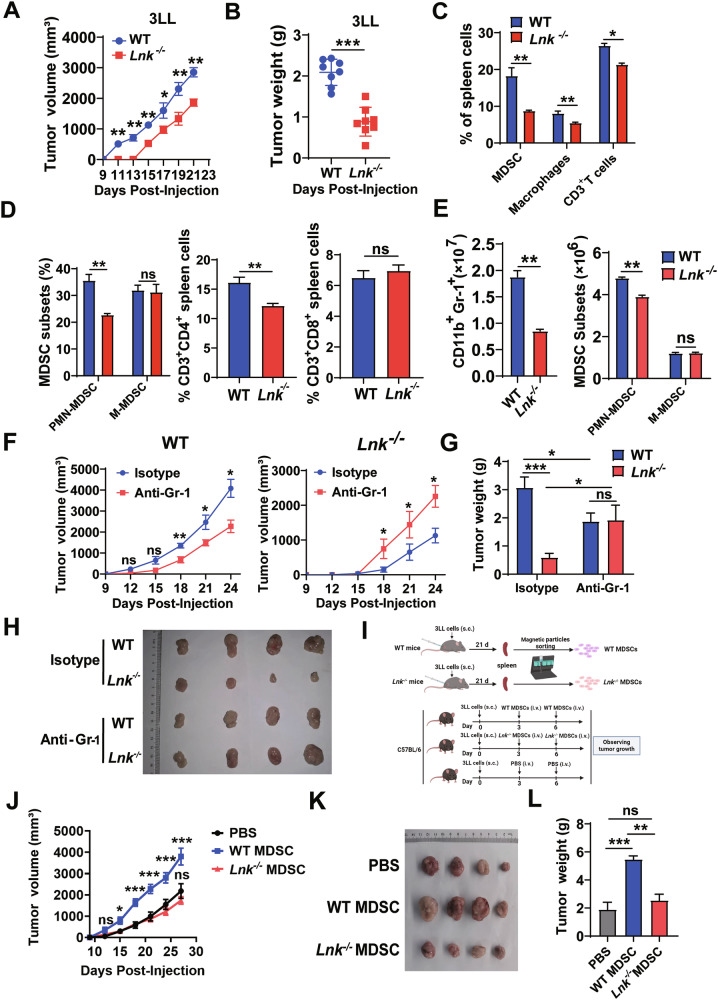
Lnk deficiency impaired MDSCs accumulation and reduced tumor progression in mice. Tumor growth kinetics (**A**) and tumor weights (**B**) in WT and *Lnk*^*–/–*^ C57BL/6 mice (n = 8 mice/group) bearing 3LL Lewis lung tumor cells as indicated. **C**, **D** Percentages of leukocyte subsets among live cells isolated from the spleens of WT and *Lnk*^*–/–*^ mice, as determined by flow cytometry 21 days after 3LL cell injection. MDSCs were CD11b^+^Gr-1^+^ (n = 4); PMN-MDSCs were CD11b^+^Ly6G^+^Ly6C^low^ (n = 4); M-MDSCs were CD11b^+^Ly6G^-^Ly6C^high^ (n = 4); macrophages were CD11b^+^F4/80^+^ (n = 4); CD3^+^ T cells were CD3^+^ (n = 4); CD4^+^ T cells were CD3^+^CD4^+^ (n = 4); and CD8^+^ T cells were CD3^+^CD8^+^ (n = 4). Representative flow cytometry results were shown in Fig. S2B. **E** Flow cytometry was performed to assess the numbers of MDSCs and subsets in the spleens of 3LL tumor-bearing mice via absolute bead counts (n = 3). Tumor growth curves (**F**), tumor weights (**G**) and representative images of tumors (**H**) from WT and *Lnk*^*–/–*^ C57BL/6 mice (n = 4) injected s.c. on day 0 with 3LL cells and that received i.p. injections of either an anti-Gr-1 antibody (RB6-8C5) or isotype control antibody once every 3 days. **I** Diagram of the experimental model of adoptive transfer. Tumor growth curves (n = 6) (**J**), representative images of tumors (**K**), and tumor weights (n = 4) (**L**) of WT mice injected s.c. on day 0 with 3LL cells, with or without subsequent i.v. injections, on days 3 and 6, of splenic WT or *Lnk*^*–/–*^ MDSCs as indicated. **M** Flow cytometric analysis of immune subsets in the tumor tissues of WT and *Lnk*^*–/–*^ mice. Representative flow cytometry results were shown in Fig. S3A–G. Tumor growth curves (n = 4) (**N**), representative images of tumors (**O**), and tumor weights (n = 4) (**P**) of WT mice injected s.c. on day 0 with 3LL cells, with or without subsequent i.v. injections, on days 3 and 6, of WT or *Lnk*^*–/–*^ MDSCs from tumor tissues. MDSCs from tumor tissues were sorted using a BD FACSAria SORP. Data are shown as means ± SEM. *P < 0.05; **P < 0.01; ***P < 0.001; ns not significant.

To investigate the potential roles of Lnk deficiency in tumorigenesis, 3LL and B16F10 cells were subcutaneously injected into WT and *Lnk*^–/–^ mice. The resultant tumors in *Lnk*^*-/*-^ mice were smaller than those in WT mice (Fig. 1A and Fig. S1A). Consistently, mice deficient in Lnk had reduced tumor weights (Fig. 1B and Fig. S1B). We next addressed whether Lnk deficiency affects immune cells to restrain tumorigenesis. To test this possibility, we analyzed the changes in the proportions of immune cells in the WT and *Lnk*^*-/*-^ mice. The percentages of leukocyte subsets, such as immature myeloid cells (IMCs), macrophages, DCs, CD3^+^ T cells, B cells, natural killer (NK) cells and Tregs, in the spleen did not differ between tumor-free WT and *Lnk*^–/–^ mice (Fig. S1C). Compared with those in the spleens of the WT controls, the percentages of MDSCs, macrophages and CD3^+^ T cells were lower in the spleens of the B16F10-bearing *Lnk*^–/–^ mice, while the percentages of DCs, B cells, NK cells and Tregs were not significantly different (Fig. S2A). Notably, the percentages of CD4^+^ T cells, CD8^+^ T cells and PMN-MDSCs were decreased in the spleens of B16F10-bearing *Lnk*^–/–^ mice, while the percentage of M-MDSCs was not significantly different (Fig. S2A). In addition, similar results were obtained in the 3LL tumor-bearing mouse model but the proportion of CD8^+^ T cells was not significantly changed in the 3LL-bearing *Lnk*^–/–^ mice compared with WT controls (Fig. S2B, Fig. 1C, D). Considering the significant difference in MDSCs, we counted the number of MDSCs and MDSC subpopulations in the spleens of tumor-bearing mice. Compared with those in the spleens of WT controls, the numbers of MDSCs and PMN-MDSCs were lower while the number of M-MDSCs was not obviously different in the spleens of B16F10/3LL-bearing *Lnk*^–/–^ mice (Fig. 1E and Fig. S2C). These data demonstrated that Lnk deletion may inhibit tumor development by reducing the accumulation of MDSCs.

To assess the effect of Lnk-deficient MDSCs on tumor growth, we depleted MDSCs in both WT and *Lnk*^–/–^ 3LL-bearing mice using an anti-Gr-1 antibody. We discovered that whereas depleting MDSCs dramatically slowed the growth of tumors in WT mice, it surprisingly increased the growth of tumors in *Lnk*^–/–^ mice (Fig. 1F–H). To confirm whether Lnk-deficient MDSCs possess antitumorigenic properties, we conducted splenic MDSCs adoptive transfer experiments in vivo. Splenic MDSCs isolated from 3LL-bearing WT or *Lnk*^–/–^ mice were injected i.v. into WT mice on days 3 and 6 after s.c. 3LL injection; a PBS injection group was used as a control (Fig. 1I). Compared with the adoptive transfer of WT splenic MDSCs, the adoptive transfer of *Lnk*^–/–^ splenic MDSCs did not promote tumor growth (Fig. 1J–L). These results suggested that Lnk deficiency in splenic MDSCs resulted in the loss of protumoral activity.

Besides, we also examined the percentages of immune cells in tumor tissues from WT and *Lnk*^–/–^ 3LL-bearing mice. Compared with the WT controls, the percentage of MDSCs and PMN-MDSCs were decreased while the percentages of M-MDSCs, macrophages, CD3^+^ T cells and CD8^+^ T cells were increased in tumor tissues of *Lnk*^–/–^ 3LL-bearing mice (Fig. 1M and Fig. S3A–G). Moreover, there were no significant differences in other tumor-infiltrating immune cells between the two groups, such as DCs, CD4^+^ T cells, B cells, NK cells and Tregs (Fig. 1M and Fig. S3H). We also sorted tumor MDSCs for adoptive transfer experiments. Similar to splenic MDSCs results, adoptive transfer of *Lnk*^–/–^ tumor MDSCs did not promote tumor growth compared with WT tumor MDSCs (Fig. 1N–P). Thus, the combined data suggested that Lnk deficiency led to reduced tumor MDSCs accumulation while loss of protumoral activity.

### Lnk deficiency blocked the immune-inhibitory activity of MDSCs

**Fig. 2:**
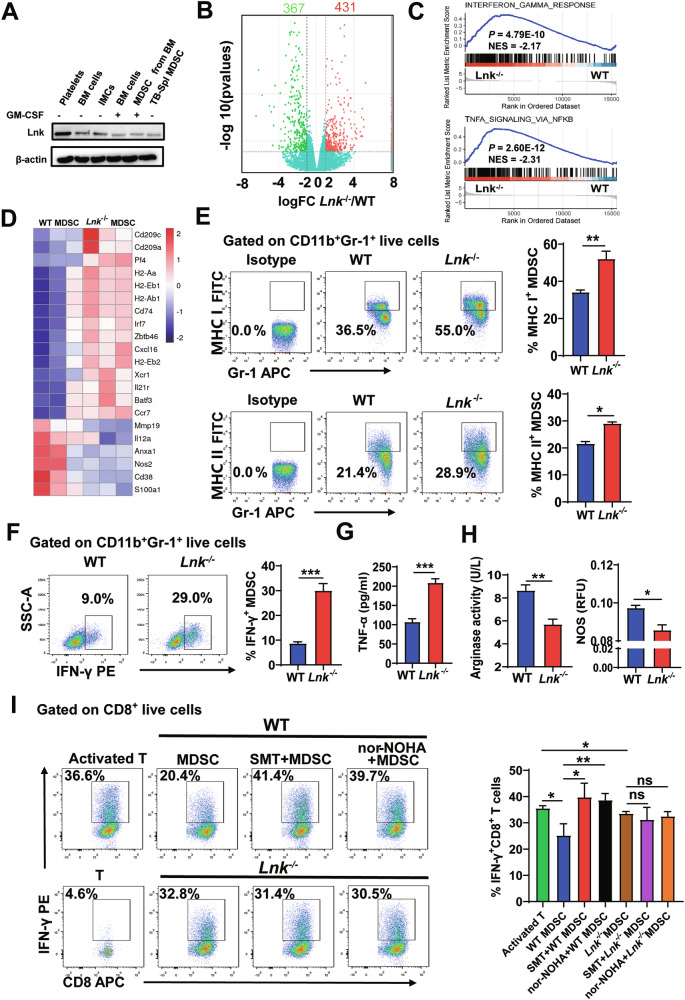
Lnk-deficient blocked the immune-inhibitory activity of MDSCs. **A** Lnk expression in BM cells, IMCs sorted from BMs, GM-CSF-stimulated BMs, MDSCs derived from GM-CSF-stimulated BMs and splenic MDSCs from tumor-bearing C57BL/6 mice. Platelets were used as positive control. **B** Volcano plots of differentially expressed genes in MDSCs isolated from the spleens of 3LL-bearing WT and *Lnk*^*–/–*^ mice (adjusted P ≤ 0.05 and fold change (FC) ≥ 1.5), n = 3. **C** Gene set enrichment analysis (GSEA) was used to assess MDSC-associated gene functional enrichment. **D** Heatmap of MDSC-associated signature genes in MDSCs isolated from the spleens of 3LL-bearing WT and *Lnk*^*–/–*^ mice. **E**, **F** The expression of MHC-I, MHC-II and IFN-γ in MDSCs (n = 4) from the spleens of WT and *Lnk*^*–/–*^C57BL/6 mice that were s.c. injected with 3LL cells was determined by flow cytometry 21 days after injection. **G** TNF-α secretion (n = 4) by MDSCs isolated from the spleens of 3LL-bearing WT and *Lnk*^*–/–*^ mice was measured by ELISA. **H** NOS expression (n = 3) in MDSCs isolated from the spleens of 3LL-bearing WT and *Lnk*^*–/–*^ mice was measured by a nitric oxide synthase assay kit. Arg-1 activity (n = 4) in MDSCs isolated from the spleens of 3LL-bearing WT and *Lnk*^*–/–*^ mice was measured by colorimetric assay. **I** Percentage of IFN-γ-expressing CD8^+^ T cells (n = 3) in cocultures of MDSCs and CD8^+^ T cells at a ratio of 1:2, as determined by flow cytometry. MDSCs were isolated from the spleens of WT and *Lnk*^*–/–*^ mice 21 days after s.c. injection of 3LL cells. CD8^+^ T cells isolated from the spleens of C57BL/6 mice were activated in vitro with anti-CD3 and anti-CD28 antibodies for 48 h before coculture; unactivated T cells were used as the control. Data are shown as means ± SEM. *P < 0.05; **P < 0.01; ***P < 0.001; ns not significant.

We found that Lnk was expressed in MDSCs derived from the BM and spleens of tumor-bearing mice (Fig. 2A). We then used RNA-seq to explore whether Lnk controls MDSC-related signature gene expression. A total of 798 genes were differentially expressed between WT and *Lnk*^–/–^ splenic MDSCs (Fig. 2B). We discovered that a substantial alteration in the gene expression patterns crucial to MDSCs function resulted from the loss of Lnk. GSEA suggested that the IFN-γ and TNF-α pathways were enriched in the *Lnk*^–/–^ MDSCs group (Fig. 2C). Compared with WT controls, the gene expression of MHC I and MHC II was higher, while the gene expression of Nos2 was lower in *Lnk*^–/–^ MDSCs (Fig. 2D). Then, we verified the results at the protein level. Compared with WT splenic MDSCs, the expression levels of MHC I, MHC II and IFN-γ were higher in the splenic MDSCs of 3LL tumor-bearing *Lnk*^–/–^ mice (Fig. 2E, F). Specifically, compared with WT splenic MDSCs, *Lnk*^–/–^ splenic MDSCs exhibited higher TNF-α levels and lower Arg-1 and NOS activity (Fig. 2G, H), with no differences in the levels of ROS and PD-L1(Fig. S4A and S4B). These results indicate that a lack of Lnk promoted the expression of immune-stimulating molecules and inhibited the expression of immune-suppressing molecules in splenic MDSCs.

MDSCs can impair productive T-cell responses, thus suppressing immune responses. Thus, we cocultured splenic MDSCs with activated CD4^+^ or CD8^+^ T cells and evaluated the immunomodulatory effects of Lnk on MDSCs. We found that the ability of MDSCs to inhibit CD4^+^ T-cell and CD8^+^ T-cell proliferation did not differ between WT splenic MDSCs and *Lnk*^-/-^ splenic MDSCs (Fig. S4C and S4D). However, *Lnk*^–/–^ splenic MDSCs showed decreased inhibition of IFN-γ secretion by CD8^+^ T cells (Fig. 2I). To explore the mechanism by which Lnk-deficient splenic MDSCs have a reduced ability to inhibit IFN-γ production by CD8^+^ T cells, we treated splenic MDSCs with either SMT (an NOS inhibitor) or nor-NOHA (an arginase inhibitor) to inhibit NOS and Arg-1 activity, respectively. We found that WT splenic MDSCs failed to inhibit IFN-γ generation by CD8^+^ T cells after the addition of SMT or nor-NOHA, while *Lnk*^–/–^ splenic MDSCs have no significant change about inhibiting IFN-γ production by CD8^+^ T cells, with or without SMT or nor-NOHA (Fig. 2I). These results suggested that the reduced ability of *Lnk*^*–/–*^ splenic MDSCs to inhibit CD8^+^ T cells may be related to the low expression of NOS and Arg-1.

To further confirm that Lnk deficiency impaired the immunosuppressive activity of MDSCs, BM-MDSCs, which were produced by cultivating BM cells in the presence of GM-CSF, were used for in vitro investigations. We reached a conclusion similar to that for the in vivo experiments: Lnk-deficient BM-derived MDSCs showed decreased NOS and Arg-1 activities and increased TNF-α and IFN-γ expression (Fig. S4E–H) and decreased the inhibition for CD8^+^ T cells to product IFN-γ (Fig. S4I). These results suggested that Lnk deficiency blocked the immune-inhibitory activity of MDSCs in a cell-intrinsic manner.

To explore whether the function of Lnk deficient tumor MDSCs was similarly altered as Lnk deficient splenic MDSCs, we investigated the expression of function factors in MDSCs from tumor tissues of WT and *Lnk*^–/–^ 3LL bearing-mice. Compared with WT controls, the protein expression of MHC I, MHC II, TNF-α and IFN-γ were higher, while the expression of Arg-1 and iNOS were lower in *Lnk*^–/–^ tumor MDSCs (Fig. S5A–F). In addition, CD8^+^T cells in the tumor tissues of *Lnk*^–/–^ mice were more proliferated and had an enhanced ability to secrete IFN-γ compared with WT mice (Fig. S5G, H). Indeed, when tested in vitro, MDSCs from tumor tissues of *Lnk*^-/-^ 3LL bearing-mice had a reduced capacity to block IFN-γ production by CD8^+^T cells (Fig. S5I), with no significant difference in inhibition of T cell proliferation (Fig. S5J), compared with those from WT controls. Thus, the combined data suggested that Lnk deficiency blocked the immune-inhibitory activity of MDSCs.

### Lnk deficiency weakened the immunosuppressive function of PMN-MDSCs

**Fig. 3:**
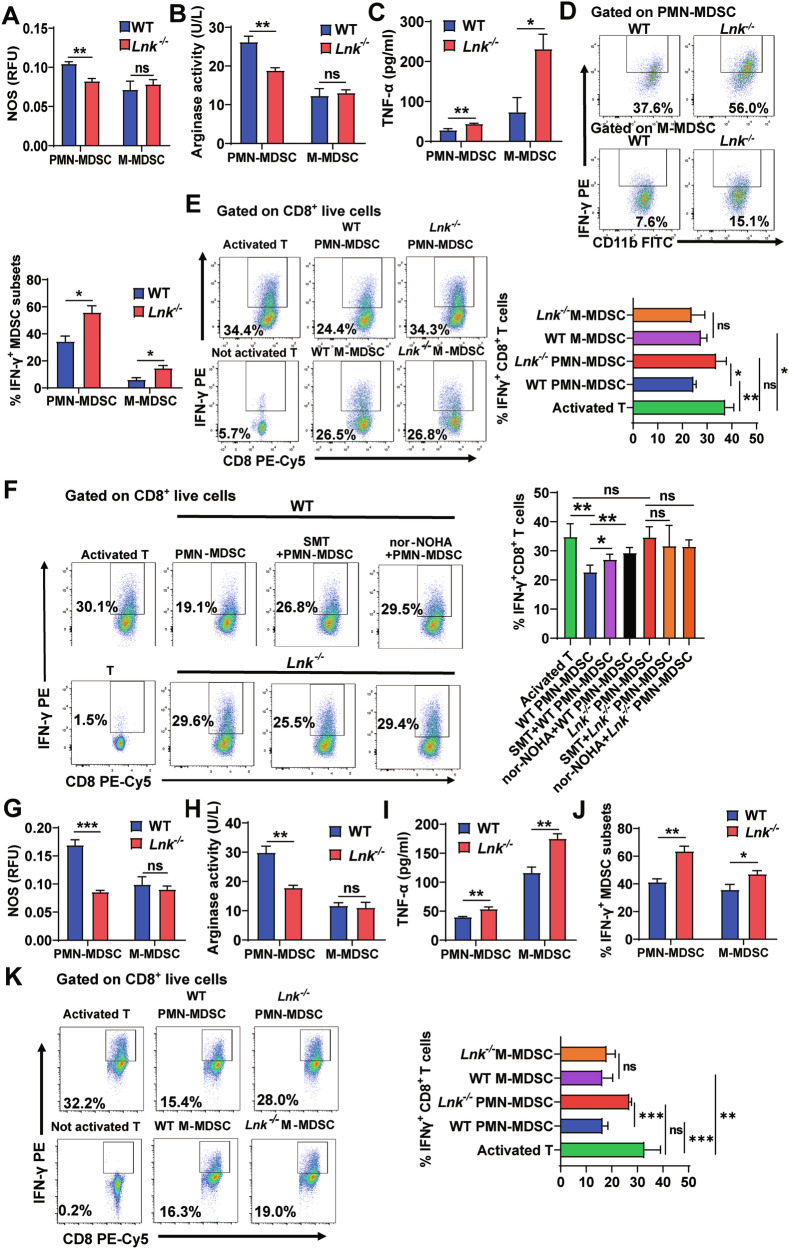
Lnk deficiency weakened the immunosuppressive function of PMN-MDSCs. 3LL lung tumor cells were subcutaneously injected into WT and *Lnk*^*–/–*^ mice. **A–F** CD11b^+^Ly6G^+^Ly6C^low^ PMN-MDSCs and CD11b^+^Ly6G^-^Ly6C^high^ M-MDSCs in splenic spleens were sorted using a BD FACSAria SORP. NOS activity (n = 4) (**A**), ARG1 activity (n = 4) (**B**), TNF-α secretion (n = 4) (**C**) and IFN-γ expression (n = 4) (**D**) in splenic MDSC subsets were measured by a nitric oxide synthase assay kit, colorimetric assay, ELISAs and flow cytometry. **E** Percentages of IFN-γ-expressing CD8^+^ T cells (n = 3) in cocultures of splenic MDSC subsets and anti-CD3/CD28-activated CD8^+^ T cells at a ratio of 1:2 respectively, as determined by flow cytometry. **F** Percentage of IFN-γ-expressing CD8^+^ T cells (n = 4) in cocultures of splenic PMN-MDSCs and anti-CD3/CD28 activated CD8^+^ T cells at a ratio of 1:2, with or without SMT or nor-NOHA, as determined by flow cytometry. **G–K** CD45^**+**^CD11b^+^Ly6G^+^Ly6C^low^ PMN-MDSCs and CD45^**+**^CD11b^+^Ly6G^-^Ly6C^high^ M-MDSCs in tumor tissues were sorted using a BD FACSAria SORP. NOS activity (n = 4) (**G**), ARG1 activity (n = 4) (**H**), TNF-α secretion (n = 4) (**I**) and IFN-γ expression (n = 4) (**J**) in tumor MDSC subsets were measured by a nitric oxide synthase assay kit, colorimetric assay, ELISAs and flow cytometry. Representative flow cytometry results were shown in Fig. S5K. **K** Percentages of IFN-γ-expressing CD8^+^ T cells (n = 4) in cocultures of tumor MDSC subsets and anti-CD3/CD28-activated CD8^+^ T cells at a ratio of 1:2 respectively, as determined by flow cytometry. Data are shown as means ± SEM. *P < 0.05; **P < 0.01; ***P < 0.001; ns not significant.

To confirm which subpopulation was affected by Lnk deficiency, we detected corresponding functional molecules in splenic MDSC subsets. We found that the activities of NOS and Arg-1 in splenic PMN-MDSCs from 3LL-bearing *Lnk*^–/–^ mice were decreased, while the expression of IFN-γ and TNF-α was increased (Fig. 3A–D). We also found that *Lnk*^–/–^ splenic PMN-MDSCs showed loss inhibition of IFN-γ expression by CD8^+^ T cells, but *Lnk*^–/–^ splenic M-MDSCs did not affect the inhibition of IFN-γ production by CD8^+^ T cells compared with that of WT splenic M-MDSCs (Fig. 3E). Besides, *Lnk*^–/–^ splenic PMN-MDSCs failed to inhibit IFN-γ production by CD8^+^ T cells with or without SMT or nor-NOHA (Fig. 3F).

The effect of Lnk on the function of tumor MDSC subsets was also investigated. The results showed that NOS and Arg-1 activity in *Lnk*^*–/–*^ tumor PMN-MDSCs were decreased, while the expression of IFN-γ and TNF-α was increased (Fig. 3G–J). Moreover, *Lnk*^-/-^ tumor PMN-MDSCs showed loss inhibition of IFN-γ expression by CD8^+^ T cells compared with WT tumor PMN-MDSCs. However, WT and *Lnk*^*–/–*^ tumor M-MDSCs both inhibited IFN-γ expression by CD8^+^T cells, and there was no significant difference between them (Fig. 3K). Thus, the combined data suggested the weakened immunosuppressive function of *Lnk*^–/–^ MDSCs was mainly due to the weakened immunosuppressive function of the PMN-MDSCs subset.

### Lnk deletion reduced the immunosuppressive function of MDSCs via ferroptosis

**Fig. 4:**
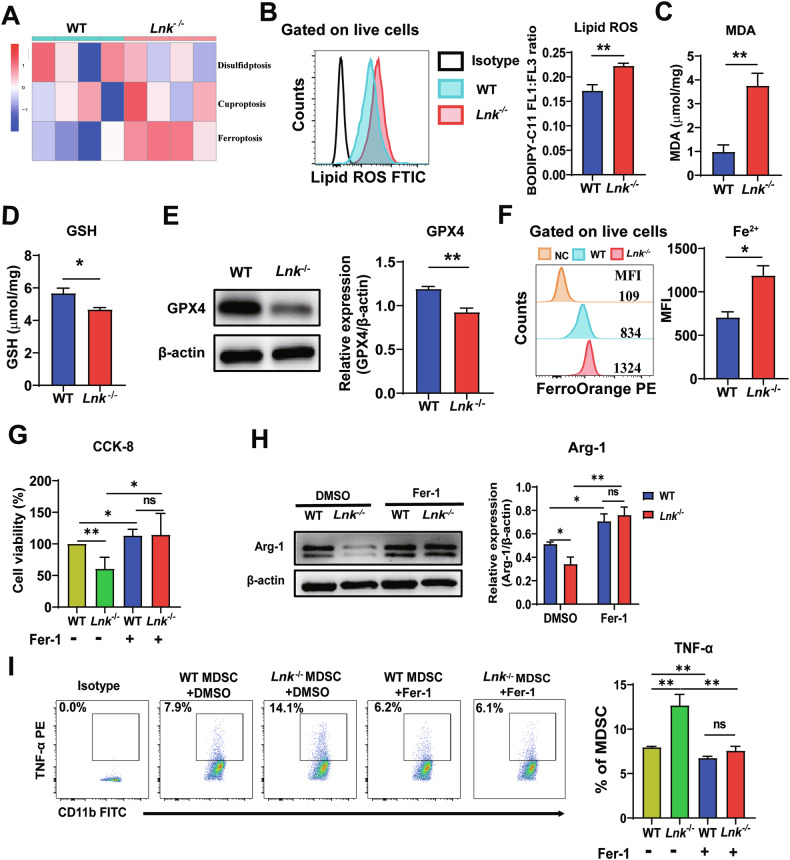
Lnk deletion reduced the immunosuppressive function of MDSCs via ferroptosis. **A** Heatmap of cell death-related genes in MDSCs isolated from the spleens of 3LL-bearing WT and *Lnk*^*–/–*^ mice, n = 4. **B** Flow cytometric analysis of lipid ROS in MDSCs isolated from the spleens of 3LL-bearing WT and *Lnk*^*–/–*^ mice, n = 4. **C**, **D** Measurement of total GSH and MDA levels in MDSCs isolated from the spleens of 3LL-bearing WT and *Lnk*^*–/–*^ mice, n = 3. **E** Western blot analysis of GPX4 in MDSCs isolated from the spleens of 3LL-bearing WT and *Lnk*^*–/–*^ mice, n = 4. **F** Fe^2+^ was measured by FerroOrange in MDSCs isolated from the spleens of 3LL-bearing WT and *Lnk*^*–/–*^ mice; the NC group received no FerroOrange, n = 3. **G** Cell viability of WT and *Lnk*^*–/–*^MDSCs from the spleens of 3LL-bearing WT and *Lnk*^*–/–*^ mice treated 24 h for with DMSO or Fer-1, n = 4. **H** Protein expression level of Arg-1 in WT and *Lnk*^*–/–*^MDSCs treated for 24 h with DMSO or Fer-1, n = 5. **I** Flow cytometric analysis of TNF-α in WT and *Lnk*^*–/–*^MDSCs from the spleens of 3LL-bearing WT and *Lnk*^*–/–*^ mice treated for 24 h with DMSO or Fer-1, n = 4. Data are shown as means ± SEM. *P < 0.05; **P < 0.01; ***P < 0.001; ns not significant.

Given previous findings that Lnk deletion leads to a reduced number of MDSCs, we first detected the cell proliferation and apoptosis of MDSCs. The results showed that compared with WT MDSCs, the cell proliferation and apoptosis of *Lnk*^*–/–*^ MDSCs had no significance (Fig. S6A, B). Moreover, there was no significant difference in the levels of pyroptosis related proteins (NLRP3, GSDMD and IL-1β) between WT and *Lnk*^-/-^ MDSCs (Fig. S6C). Next, through an in-depth analysis of the special mode of cell death via RNA-seq, we found that there was no significant difference in disulfidptosis or cuproptosis between *Lnk*^*–/–*^ MDSCs and WT MDSCs; however, ferroptosis was significantly increased in *Lnk*^–/–^ MDSCs (Fig. 4A). We hypothesized that Lnk deletion could modulate MDSCs through the ferroptosis pathway. We found that the levels of lipid ROS and MDA were greater in *Lnk*^-/-^ MDSCs than in WT MDSCs, whereas ferroptosis-inhibiting genes such as GSH and GPX4 were significantly downregulated in *Lnk*^–/–^ specimens (Fig. 4B–E). Compared with WT MDSCs, the Fe^2+^ content in *Lnk*^–/–^ MDSCs was greater (Fig. 4F). According to the findings described above, we concluded that ferroptosis was induced in *Lnk*^–/–^ MDSCs.

To clarify the role of ferroptosis in *Lnk*^–/–^ MDSCs, we pretreated the splenic MDSCs of 3LL tumor-bearing WT*/Ln*k^–/–^ mice with 2 μM Fer-1 (a ferroptosis inhibitor) and then assessed the functional molecular expression in MDSCs. The viability of *Lnk*^*–/–*^ MDSCs was lower than that of WT MDSCs in the untreated group; however, after Fer-1 treatment, the viability of WT MDSCs and *Lnk*^–/–^ MDSCs significantly improved, and the viability did not significantly differ between the two groups (Fig. 4G). As shown in Fig. 4H, I, Arg-1 expression was significantly lower and TNF-α expression was greater in *Lnk*^–/–^ MDSCs than in WT MDSCs, and these changes were significantly reversed by Fer-1 treatment. These results revealed that Lnk deficiency inhibited the accumulation and immunosuppressive function of MDSCs via ferroptosis.

### IKE treatment inhibited tumor growth and reduced the immunosuppressive function of MDSCs in *Lnk*^*–/–*^ mice

Imidazole ketone erastin (IKE) is an effective and selective ferroptosis activator. To investigate the regulatory effect of Lnk deficiency mediated ferroptosis on MDSCs function in vivo, we evaluated the tumor growth kinetics and tumor weight of WT and *Lnk*^–/–^ 3LL tumor-bearing mice with or without IKE treatment. Daily IKE treatment was initiated on day 9 post-tumor inoculation (Fig. S7A). Compared with the DMSO-treated WT mice, there were no difference in tumor growth and tumor weight were seen in the IKE-treated WT mice, while IKE treatment further significantly retarded tumor growth in *Lnk*^–/–^ 3LL tumor-bearing mice (Fig. S7B, C). Ferroptosis is accompanied by peroxidation of polyunsaturated species of phosphatidylethanolamine (PE) to hydroperoxy- (-OOH) derivatives, which act as death signals, such as PE-16:0/22:4-OOH and PE-18:0/20:5-OOH. Through targeted phospholipid analysis using the HPLC-MS/MS system of tumor MDSCs, we found that in the DMSO-treated control, PE-16:0/22:4-OOH and PE-18:0/20:5-OOH were higher in *Lnk*^–/–^ tumor MDSCs than in WT tumor MDSCs (Fig. S7D). After IKE treatment, the levels of PE-16:0/22:4-OOH and PE-18:0/20:5-OOH increased in *Lnk*^–/–^ tumor MDSCs and WT tumor MDSCs, though the increase was more significant in *Lnk*^–/–^ tumor MDSCs (Fig. S7D). These results indicated that although IKE triggered MDSCs ferroptosis in immunocompetent mice, it failed to inhibit tumor growth. In contrast, Lnk deficiency combined with IKE treatment could significantly attenuate tumor development.

We next asked whether the IKE treatment affected the immunosuppressive of MDSCs in tumor tissue. By flow cytometry, we detected the expression of functional molecules and the suppressive effects on T cells of tumor MDSCs treated without/with IKE. We observed IKE treatment markedly upregulated TNF-α level and downregulated Arg-1 level in WT and *Lnk*^-/-^ tumor MDSCs (Fig. S7E, F). Meanwhile, IKE treatment attenuated the immunosuppressive capacity of MDSCs from both WT and *Lnk*^-/-^ tumor-bearing mice on T cells (Fig. S7G). These effects were more pronounced when Lnk deficiency was combined with IKE treatment. Collectively, these data demonstrate that IKE treatment inhibited tumor growth and reduced the immunosuppressive function of MDSCs in *Lnk*^-/-^ mice.

### Lnk deficiency inhibited the immunosuppressive activity of MDSCs through Flt3-mediated ferroptosis

**Fig. 5:**
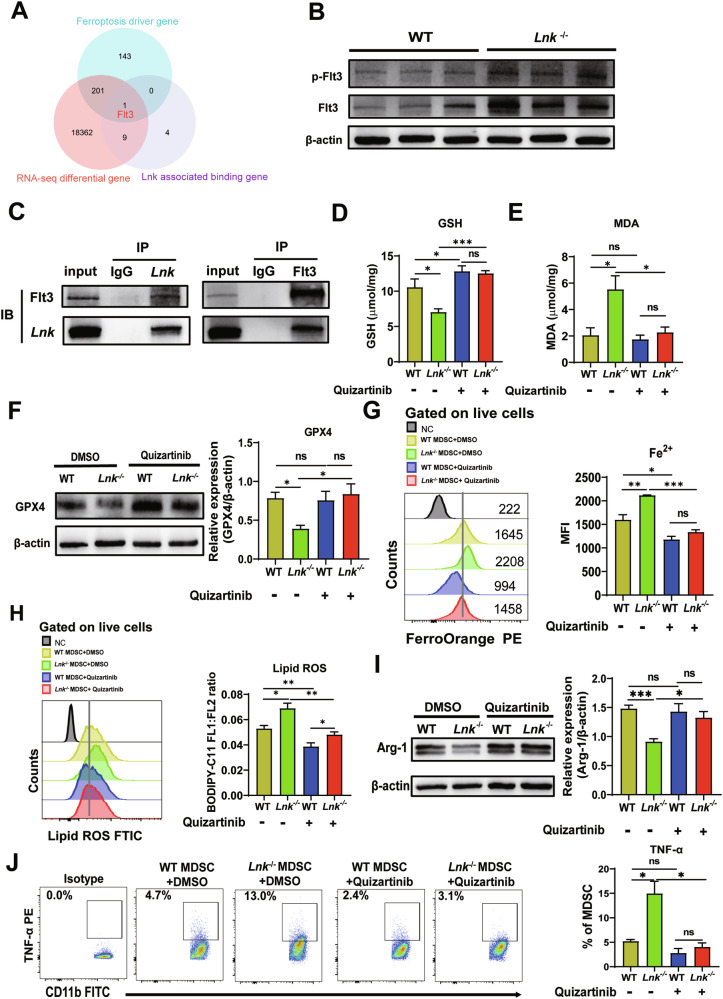
Lnk deficiency inhibited the immunosuppressive activity of MDSCs through Flt3-mediated ferroptosis. **A** Wayne map of Lnk-associated binding genes, differentially expressed genes and ferroptosis driver genes in MDSCs isolated from the spleens of 3LL-bearing WT and *Lnk*^*–/–*^ mice, n = 4. **B** Western blot analysis of Flt3 protein expression in MDSCs isolated from the spleens of 3LL-bearing WT and *Lnk*^*–/–*^ mice, n = 3. **C** Immunoprecipitation analysis of *Lnk* and Flt3 interaction in MDSCs. **D**, **E** Measurement of total GSH and MDA levels in WT and *Lnk*^*–/–*^ MDSCs treated with DMSO or quizartinib, n = 4. **F** Western blot analysis of GPX4 protein expression in WT and *Lnk*^*–/–*^ MDSCs treated with DMSO or quizartinib, n = 3. **G** Fe^2+^ in WT and *Lnk*^*–/–*^ MDSCs treated with DMSO or quizartinib was assessed using FerroOrange, n = 4. **H** Flow cytometric analysis of lipid ROS in WT and *Lnk*^*–/–*^ MDSCs treated with DMSO or quizartinib, n = 4. **I** Western blot analysis of Arg-1 protein expression in WT and *Lnk*^*–/–*^ MDSCs treated with DMSO or quizartinib for 24 h, n = 4. **J** Flow cytometric analysis of TNF-α expression in WT and *Lnk*^*–/–*^ MDSCs treated with DMSO or quizartinib for 24 h, n = 4. Data are shown as means ± SEM. *P < 0.05; **P < 0.01; ***P < 0.001; ns not significant.

We next explored the mechanism underlying *Lnk* deletion-induced ferroptosis in MDSCs. We identified an intersecting gene Flt3 through RNA-seq analyses of differentially expressed genes, ferroptosis-driving genes and Lnk-associated binding genes (Fig. 5A). Flt3 is a receptor tyrosine kinase with important roles in cell survival and differentiation, and its expression and phosphorylation are inhibited by direct binding to Lnk [34–36]. Consistent with these findings, the expression and phosphorylation of Flt3 were higher in *Lnk*^-/-^ MDSCs than in WT MDSCs (Fig. 5B). Coimmunoprecipitation revealed that Lnk can indeed bind to Flt3 in MDSCs (Fig. 5C). To explore whether Flt3 can regulate ferroptosis, we examined ferroptosis indices in MDSCs treated with 5 μM quizartinib (an Flt3 inhibitor). The results showed that MDSCs from WT and *Lnk*^-/-^ mice pretreated with quizartinib had increased GSH levels (Fig. 5D). The increase in MDA levels and decrease in GPX4 expression in *Lnk*^*–/–*^ MDSCs were also reversed after pretreatment with the inhibitor (Fig. 5E, F). Furthermore, the levels of Fe^2+^ and lipid ROS in WT and *Lnk*^–/–^ MDSCs decreased after treatment with quizartinib, and they were more significantly reduced in *Lnk*^–/–^ MDSCs (Fig. 5G, H). Overall, these results indicate that Lnk defects may promote the phosphorylation of Flt3, which induces ferroptosis in MDSCs.

To confirm the contribution of Flt3-mediated ferroptosis to the immunosuppressive activity of MDSCs, we assessed the expression of immunosuppressive and immune-activating molecules in WT and *Lnk*^*–/–*^ MDSCs treated with quizartinib. We found that Arg-1 expression was lower in *Lnk*^*–/–*^ MDSCs than in WT MDSCs but was greater in *Lnk*^*–/–*^ MDSCs pretreated with quizartinib (Fig. 5I). We also found that *Lnk*^*–/–*^ MDSCs secreted more TNF-α than did WT MDSCs; however, this effect was eliminated after treatment with quizartinib (Fig. 5J). Collectively, these data suggest that Lnk deficiency impairs the immunosuppressive activity of MDSCs through Flt3-mediated ferroptosis.

### Flt3 promotes the expression of Alox12 through the Stat1/IRF1 axis to regulate the immunosuppression of MDSCs

**Fig. 6:**
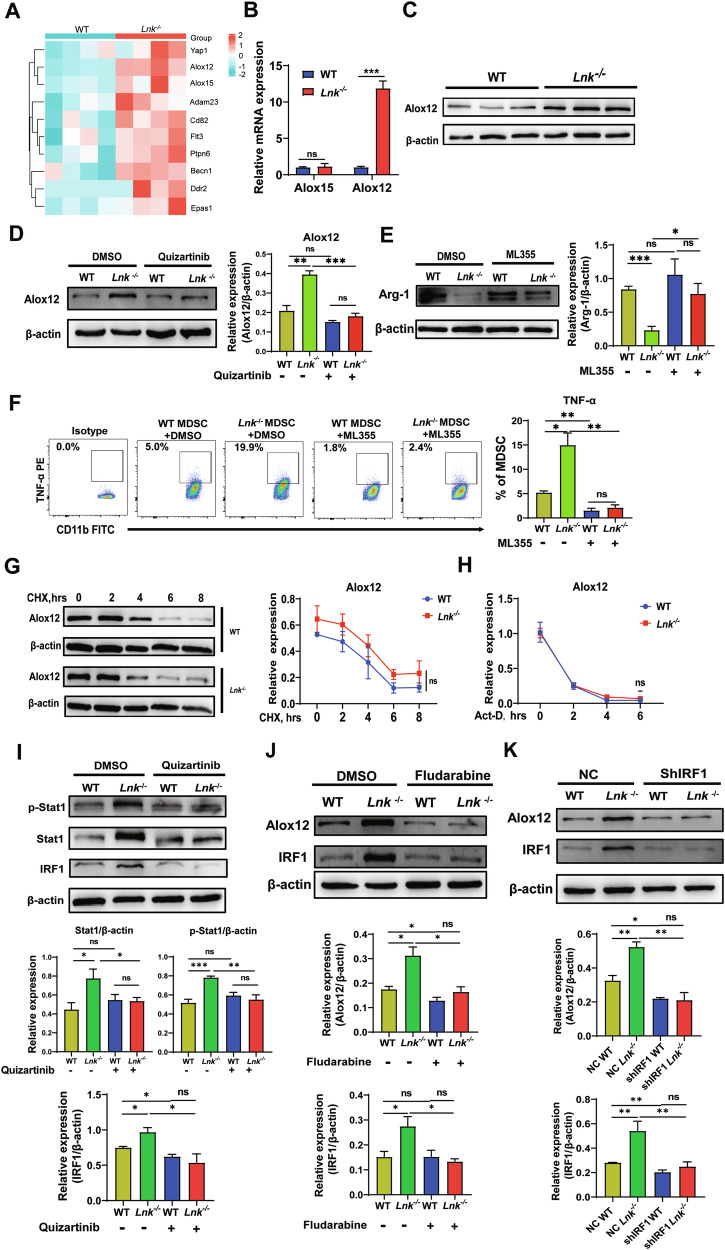
Flt3 promotes the expression of Alox12 through the Stat1/IRF1 axis to regulate the immunosuppression of MDSCs. **A** Heatmap of differentially expressed genes and ferroptosis driver genes in MDSCs isolated from the spleens of 3LL-bearing WT and *Lnk*^*–/–*^ mice, n = 4. **B** RT‒qPCR analysis of Alox12/Alox15 gene expression in MDSCs isolated from the spleens of 3LL-bearing WT and *Lnk*^*–/–*^ mice, n = 4. **C** Western blot analysis of Alox12 protein expression in MDSCs isolated from the spleens of 3LL-bearing WT and *Lnk*^*–/–*^ mice, n = 3. **D** Protein expression level of Alox12 in WT and *Lnk*^*–/–*^ MDSCs treated with DMSO or quizartinib, n = 3. **E** Western blot analysis of Arg-1 protein expression in WT and *Lnk*^*–/–*^ MDSCs treated with DMSO or ML355 for 24 h, n = 4. **F** Flow cytometric analysis of TNF-α expression in WT and *Lnk*^*–/–*^ MDSCs treated with DMSO or ML355 for 24 h, n = 4. **G** WT and *Lnk*^*–/–*^ MDSCs were treated with CHX, and the cells were collected at 0 h, 2 h, 4 h, 6 h, and 8 h to assess Alox12 protein expression. **H** WT and *Lnk*^*–/–*^ MDSCs were treated with Act-d, and the cells were collected at 0 h, 2 h, 4 h, and 6 h. RT‒qPCR was used to assess the mRNA levels of Alox12. **I** Western blotting was used to measure the protein expression levels of Stat1, p-Stat1 and IRF1 in WT and *Lnk*^*–/–*^ MDSCs treated with DMSO or quizartinib for 24 h, n = 4. **J** Western blotting was used to measure the protein expression levels of Alox12 and IRF1 in WT and *Lnk*^*–/–*^ MDSCs treated with DMSO or fludarabine for 24 h, n = 3. **K** Protein expression levels of Alox12 and IRF1 in NC and shIRF1 WT and *Lnk*^*–/–*^ MDSCs, n = 3. Data are shown as means ± SEM. *P < 0.05; **P < 0.01; ***P < 0.001; ns not significant.

To explore how Flt3 regulates ferroptosis, we analyzed heatmaps of differentially expressed genes and ferroptosis driver genes identified in WT and *Lnk*^*–/–*^ MDSCs via RNA-seq (Fig. 6A). We screened two noteworthy genes (Alox12 and Alox15) for gene level validation by RT‒qPCR. We found that, compared with WT MDSCs, there was no significant difference in the expression of Alox15 in *Lnk*^–/–^ MDSCs, but Alox12 expression was higher in *Lnk*^-/-^ MDSCs at the mRNA level (Fig. 6B). Moreover, the protein expression level of Alox12 was also significantly increased in *Lnk*^–/–^ MDSCs (Fig. 6C). Alox12 causes the direct peroxidation of PUFAs to lethal lipid ROS, which then accumulate and trigger ferroptosis. Emerging research has shown that Flt3 inhibitors may reduce cell death by preventing the oxidation of PUFAs [37]. Based on previous results, we speculated that Flt3 might regulate ferroptosis via Alox12. First, we detected Alox12 protein expression levels in MDSCs treated with 5 μM quizartinib. We found that Alox12 expression was significantly lower in the treated group than in the untreated group (Fig. 6D). This finding suggest that Lnk binds to Flt3 and inhibits its expression and phosphorylation, which may reduce the expression of Alox12 and inhibit ferroptosis in MDSCs.

Next, we assessed Arg-1 and TNF-α expression in WT and *Lnk*^*–/–*^ MDSCs treated with ML355 (an Alox12 inhibitor). We found that the decrease in Arg-1 expression in *Lnk*^*–/–*^ MDSCs was reversed after pretreatment with ML355 (Fig. 6E). The levels of TNF-α in WT and *Lnk*^–/–^ MDSCs decreased after treatment with ML355, and they were significantly decreased in *Lnk*^–/–^ MDSCs (Fig. 6F). Together, these results affirm that Lnk deficiency impairs the immunosuppressive activity of MDSCs through Flt3/Alox12-mediated ferroptosis.

We investigated how Flt3 regulates the expression of Alox12. Increased protein expression may be achieved through reduced protein degradation, increased transcription, and increased RNA stability. First, we treated WT and *Lnk*^*–/–*^ MDSCs with cycloheximide (CHX, a protein synthesis inhibitor) and actinomycin D (Act-d, a DNA transcription and replication inhibitor) and harvested the cells at 0 h, 2 h, 4 h, 6 h, and 8 h. The protein and mRNA expression levels of Alox12 were analyzed. We found that there was no significant difference in the protein degradation rate or RNA stability of Alox12 between the *Lnk*^*–/–*^ group and the WT group (Fig. 6G, H). Therefore, we hypothesized that the change in Alox12 protein expression is caused by a change in transcription level. Using the PROMO database, we screened interferon regulatory factor 1 (IRF1) as a transcription factor for Alox12. IRF1 has been identified as a critical factor in mediating inflammatory cell death. In addition, previous studies have indicated that Stat1 works upstream of IRF1 and that Stat1 phosphorylation can lead to the recruitment of IRF1 [38, 39]. We hypothesized that Flt3 activates Stat1, which is phosphorylated and recruits IRF1 to initiate the transcription of Alox12. As shown in Fig. 6I, compared with that in WT MDSCs, the protein expression and phosphorylation of Stat1 in *Lnk*^*-/-*^ MDSCs was higher, but the the protein expression and phosphorylation levels decreased significantly after Flt3 was inhibited. The same change occurred for the downstream transcription factor IRF1 after treatment with quizartinib (Fig. 6I). Next, we treated two groups of cells with fludarabine (a Stat1 inhibitor). Consistently, after Stat1 was inhibited, the expression of Alox12 and IRF1 decreased (Fig. 6J). To determine the regulatory effect of IRF1 on Alox12, IRF1 was knocked down in primary mouse MDSCs. As expected, after IRF1 knockdown, Alox12 expression was significantly reduced in the WT and *Lnk*^*–/–*^MDSCs (Fig. 6K). Taken together, these data suggest that Flt3 may stimulate Alox12 expression via the Stat1/IRF1 axis.

### Lnk is highly expressed in MDSCs from lung cancer patients and is associated with the function of human MDSCs

**Fig. 7:**
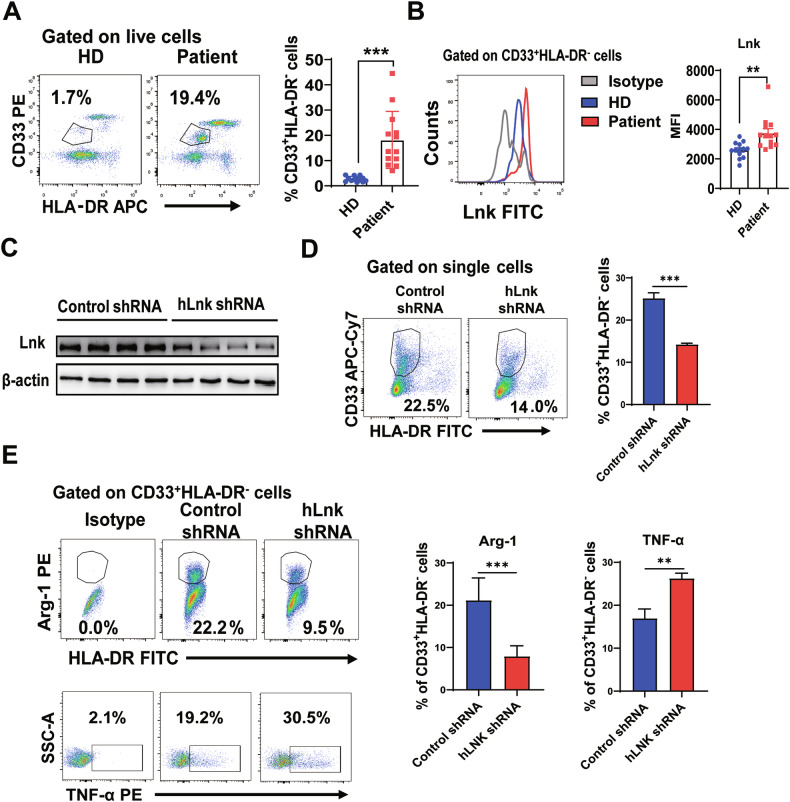
Lnk is highly expressed in MDSCs from lung cancer patients and is associated with the function of human MDSCs. **A** Percentages of the indicated HLA-DR^−^CD33^+^ cells among the PBMCs of healthy donors (HDs) and lung cancer patients, as determined by flow cytometry. HD, n = 13; patient, n = 13. **B** Using intracellular labeling to stain for Lnk expression in HLA-DR^−^CD33^+^ MDSCs among PBMCs from lung cancer patients and HDs by flow cytometry, n = 13. **C** PBMCs from lung cancer patients were cultured in the presence of human GM-CSF and IL-6 for 2 days, infected with control or human Lnk (hLnk) shRNA-encoding lentiviruses and cultured for 2 additional days with GM-CSF and IL-6. Lnk expression in HLA-DR^−^CD33^+^ cells that was sorted from PBMCs was assessed by Western blotting, n = 4. **D** Percentages of PBMC-derived HLA-DR^−^CD33^+^ cells were determined by flow cytometry, n = 5. **E** The expression levels of Arg-1 (n = 6) and TNF-α (n = 8) in HLA-DR^−^CD33^+^ cells were determined by flow cytometry. Data are shown as means ± SEM. *P < 0.05; **P < 0.01; ***P < 0.001; ns, not significant.

Patients with diverse cancer types have been shown to harbor HLA-DR^−^CD33^+^ MDSCs with varying properties and immune-suppressive traits. We initially examined the proportions of MDSCs from lung cancer patients. The proportions of MDSCs among the PBMCs of cancer patients were notably greater than those among PBMCs from healthy individuals (Fig. 7A). We next assessed Lnk expression in MDSCs in the peripheral blood of healthy donors and lung cancer patients. As shown in Fig. 7B, compared with healthy donors, Lnk expression in MDSCs from lung cancer patients was higher. In order to ascertain the function of Lnk in human MDSCs, PBMCs from lung cancer patients were cultured in the presence of human GM-CSF and IL-6 for two days before being infected with control or human Lnk (hLnk) shRNA-encoding lentiviruses. We then sorted HLA-DR^−^CD33^+^ cells and assessed Lnk expression. MDSCs treated with Lnk shRNA expressed markedly less Lnk protein (Fig. 7C). However, the percentage of MDSCs was lower in the hLnk shRNA-treated PBMCs than in the control shRNA-treated PBMCs (Fig. 7D). Compared with control group, hLnk shRNA-treated MDSCs exhibited significantly lower expression of Arg-1 but higher expression of TNF-α (Fig. 7E). These results show that Lnk can promote the accumulation and immunosuppressive phenotype of human MDSCs.

## Discussion

MDSCs are strongly associated with the development of tumors [3, 9, 40]. Multiple findings suggest that clearing MDSCs in vivo with anti-GR-1 neutralizing antibodies can enhance the efficacy of cancer immunotherapy [41, 42]. Studying the molecular mechanisms and regulatory pathways that impact the immunosuppressive role of MDSCs may lead to more precise and targeted approaches for preventing and treating tumors. In this study, we demonstrate that Lnk plays a critical role in regulating immunosuppressive activity of MDSCs and elucidate underlying mechanisms. Lnk deficiency reduced the number of MDSCs and impaired immunosuppressive activity of MDSCs via ferroptosis. The development of Lnk inhibitors and/or ferroptosis agonists may broaden the MDSCs-centered tumor immunotherapy strategy.

Lnk may directly control the development of tumors within tumor cells or indirectly influence tumor progression through immune cells. Lnk expression in various tumor cells promotes cancer cell proliferation and migration and therefore facilitates tumorigenesis, such as in melanoma, ovarian cancer, and breast cancer [43–45]. In addition, Lnk is also expressed in hematopoietic stem cells and has an inhibitory effect on hematologic tumors, such as myeloproliferative tumors and acute lymphoblastic leukemia [13, 46–48]. Studies on the regulatory role of Lnk in immune cells are insufficient and have focused mainly on inflammatory diseases. Lnk can prevent inflammatory CD8^+^ T cells proliferation and maintain intestinal homeostasis [21]. Lnk deletion has been shown to promote DCs activation and differentiation of effector T cells [22]. In our work, we specifically found that Lnk was essential for the immunosuppressive function of MDSCs and indirectly regulated tumor progression. Lnk deficiency could reduce the ability of MDSCs to inhibit IFN-γ production by CD8^+^T cells, but did not affect the ability of MDSCs to inhibit CD8^+^T cell proliferation. Our experiment did not rule out that other cells may affect the function of CD8^+^T cells in *Lnk*^*–/–*^ mice tumor tissues, for example macrophage, or that Lnk has a direct effect on CD8^+^T cells but rather revealed the critical role of Lnk in MDSCs. In the future, Lnk may be used as a therapeutic target for MDSCs, which can effectively curb immunosuppression.

Lnk can regulate the function of immune cells in various ways, for example inhibiting the secretion of cytokines such as IFN-γ and TNF-α, and inhibiting the proliferation of macrophages and T cells [21, 22]. However, whether Lnk can regulate the function of immune cells through ferroptosis remain poorly understood. In recent years, ferroptosis in immune cells has received increasing attention [29, 49, 50]. Ferroptosis is thought to be a novel player in the TME that repolarizes immunosuppressive cells to exert direct antitumor effects [51]. For example, macrophages have been found to undergo ferroptosis that promoted M1 phenotype polarization and thus limited HCC tumor growth [52]. Ferroptosis in MDSCs can be induced by reduced GSH or the accumulation of lipid ROS [31]. Asah2 has been shown to inhibit ferroptosis in MDSCs via the p53–Hmox1 pathway and promote MDSCs accumulation, accelerating tumor growth [31]. These findings were consistent with our results. We found that Lnk deficiency caused ferroptosis in MDSCs through the accumulation of lipid ROS, thereby blocking the immunosuppression of MDSCs and suppressing tumor growth. However, it has also been reported that PMN-MDSCs undergo ferroptosis via Alox12/15, which renders them more immunosuppressive [53]. They demonstrated that MDSCs employ a coordinated Alox12/Alox15 mechanism to induce ferroptosis, thereby enhancing their immunosuppression. This was evidenced by increased PGE2 production in MDSCs and concomitant impairment of inhibiting T cell proliferation. Their results showed that IKE treatment promoted ferroptosis and the accumulation of lipid peroxidation-related PE species PE-36:4-OOH and PE-18:0/20:4-OOH and slightly increased CT26 tumor growth. However, in our study, Lnk-mediated ferroptosis was only regulated by Alxo12 but not Alox15. In addition, in our system, the impaired immunosuppressive capacity of MDSCs was mainly manifested by decreased Arg-1 expression, increased TNF-α expression, and impaired ability to suppress IFN-γ secretion by T cells. Lnk-mediated ferroptosis did not regulate PEG2 secretion and T cell proliferation inhibition by MDSCs. Moreover, PE species accumulation was increased in *Lnk*^*–/–*^ tumor MDSCs after IKE treatment, mainly PE-16:0/22:4-OOH and PE-18:0/20:5-OOH, but not PE-36:4-OOH and PE-18:0/20:4-OOH. Consistent with their results that induction of ferroptosis does not prevent tumor growth in immunocompetent mice [53]. Surprisingly, Lnk depletion combined with IKE treatment further slowed tumor growth. Thus, the different mechanisms of MDSCs immunosuppression mediated by ferroptosis may account for our opposite conclusions. Alternatively, this discrepancy may also stem from differences in our model systems. They used an orthotopic tumor model, whereas our study used a subcutaneously transplanted tumor model. Of course, these interesting results gave us pause for thought and were worth exploring in depth in the future.

**Fig. 8:**
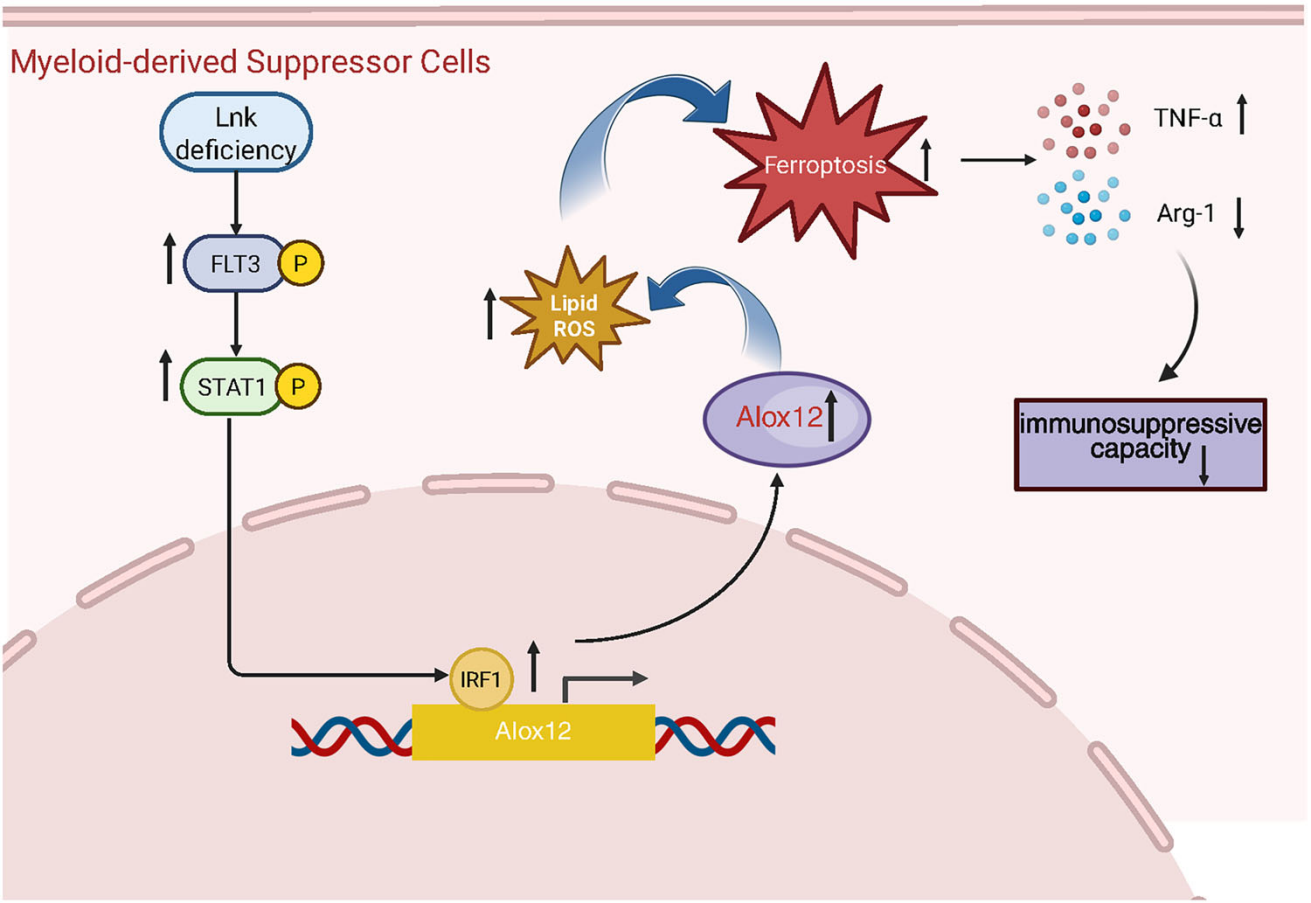
The mechanism diagram of Lnk regulating the function of MDSCs. Lnk deficiency attenuated the immunosuppressive capacity of MDSCs, mainly manifested as a decrease in Arg-1 expression and an increase in TNF-α expression. Mechanistically, Lnk deficiency impaired the function of MDSCs via Flt3/STAT1/IRF1/Alox12-mediated ferroptosis.

Flt3 mutations increase the sensitivity of acute myeloid leukemia cells to ferroptosis by triggering lipid oxidative stress [54–57]. However, the specific mechanism by which Flt3 regulates ferroptosis due to lipid peroxidation is unclear. Our evidence suggested that Flt3, which binds to Lnk, regulates ferroptosis, confirming the pivotal role of Flt3 as a link between ferroptosis and Lnk. Flt3 inhibitors have been shown to prevent ROS generation and lipid peroxidation in neuronal cells [58]. Our evidence suggested that the suppression of Flt3 inhibited ferroptosis by reducing the accumulation of lipid peroxidation ROS in MDSCs. Mechanistically, we further found that Flt3 regulated the expression of Alox12 through the Stat1/IRF1 axis, leading to the accumulation of lipid ROS, which affected ferroptosis (Fig. 8). This suggested that Flt3 was a promising target gene for activating ferroptosis in MDSCs to exert anti-tumor immunity. However, the reliability of the use of IRF1 as an Alox12 transcription factor should be further verified.

Previous studies have shown that MDSCs are present in the peripheral blood of cancer patients, infiltrate solid tumors and are closely related to malignant tumor stage and the survival time of cancer patients [59]. Further analysis of the clinical and functional characteristics of Lnk in lung cancer revealed that Lnk expression in MDSCs from lung cancer patients was greater. Knocking down Lnk in human MDSCs increased TNF-α secretion and decreased Arg-1 expression. Future studies should explore whether Lnk plays a crucial role in regulating the function of MDSCs in lung cancer patients.

In conclusion, Lnk deficiency inhibited the suppressive effect of MDSCs through ferroptosis. This effect was essential for the development of tumors, as the absence of Lnk significantly slowed tumor progression. Therefore, focusing on Lnk is a novel approach to cancer immunotherapy.

## Supplementary information

Original western blots.

Supplementary Table S1 Key Reagent

Supplemental materials

## Data Availability

All relevant data within the paper will be made available on request.
