# Supplementary material for: Lnk deficiency attenuates the immunosuppressive capacity of MDSCs via ferroptosis to suppress tumor development
Source: Cell Death Dis. 2025 Aug 12;16(1):610. doi: 10.1038/s41419-025-07948-8 (PMC12343911; doi:10.1038/s41419-025-07948-8)

Figure 2A

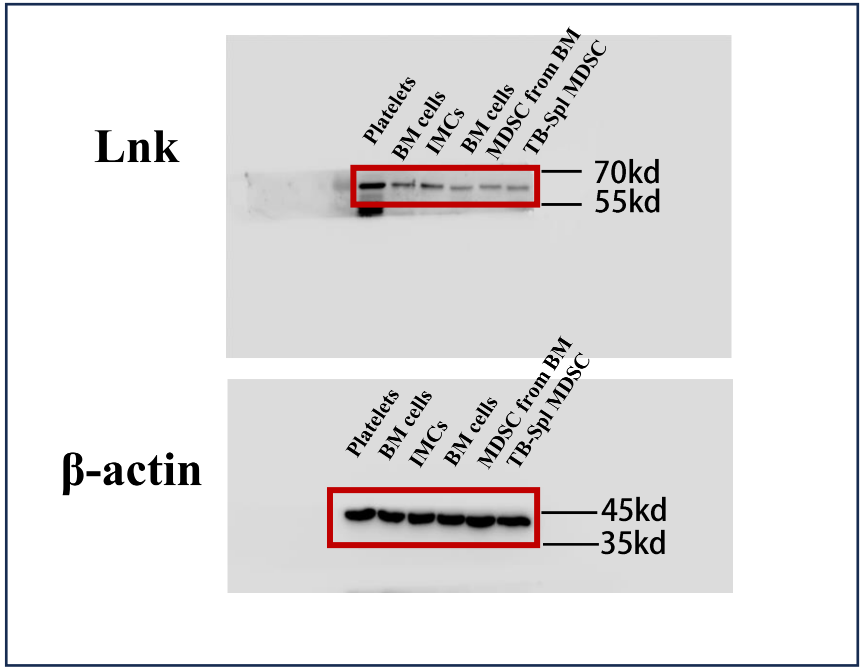

Figure 4E

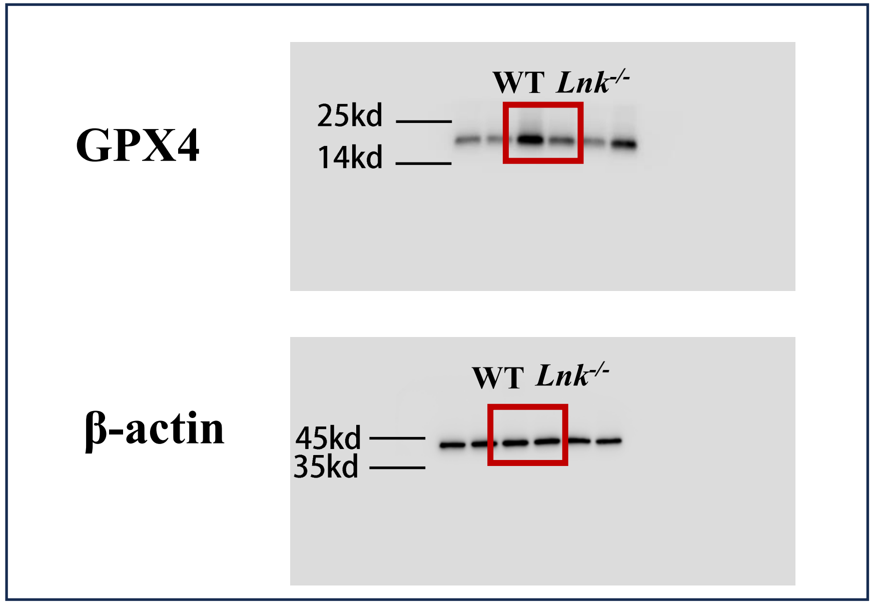

Figure 4H

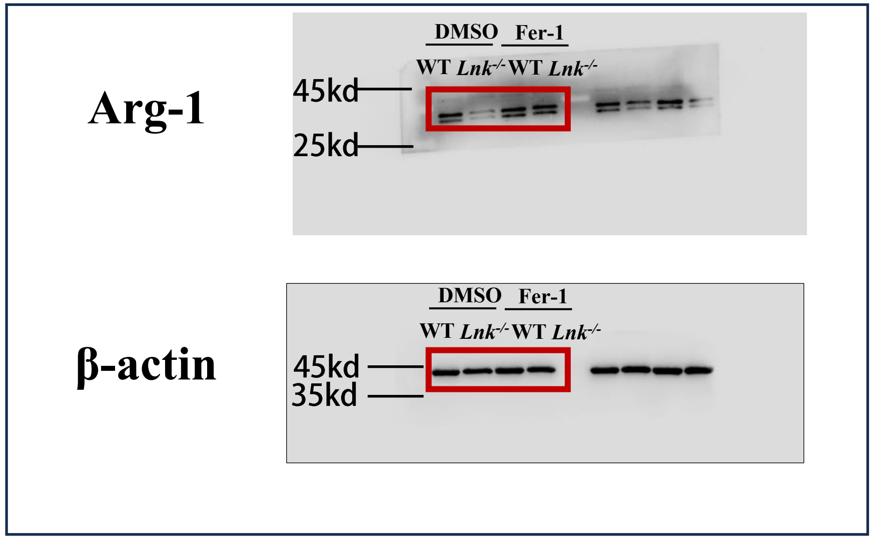

Figure 5B

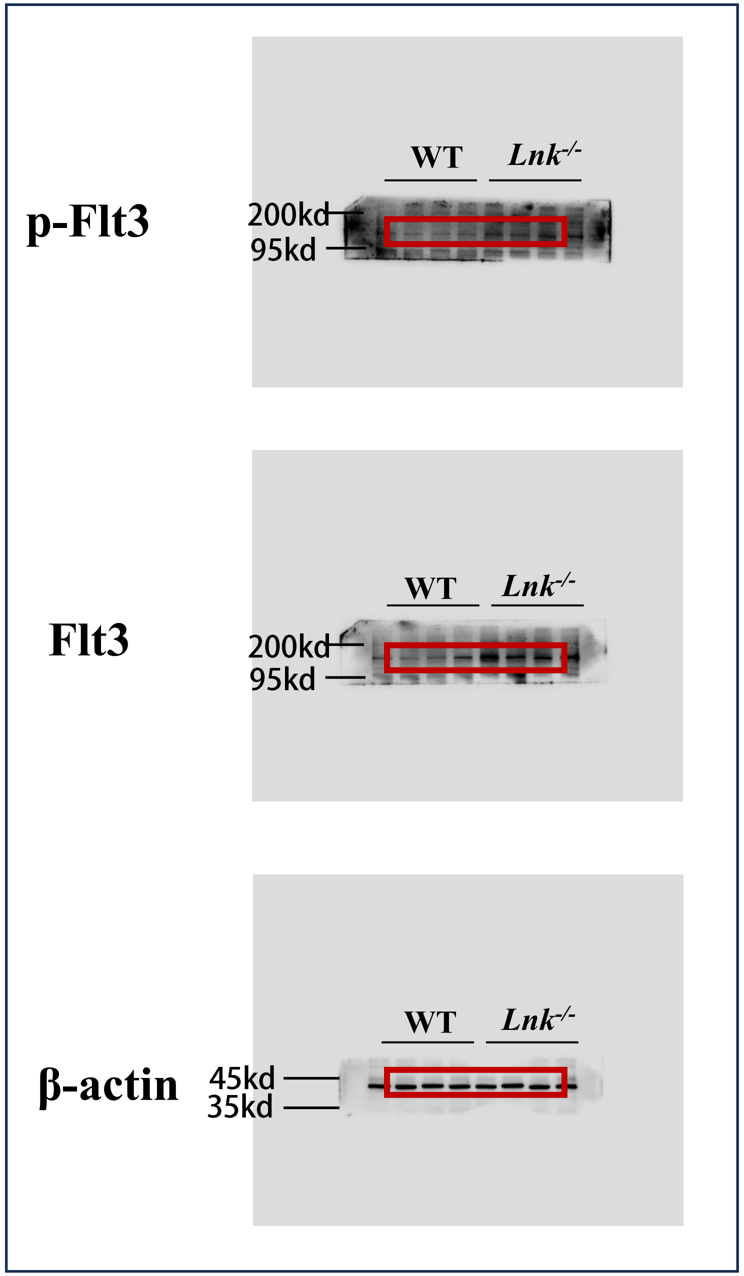

Figure 5C

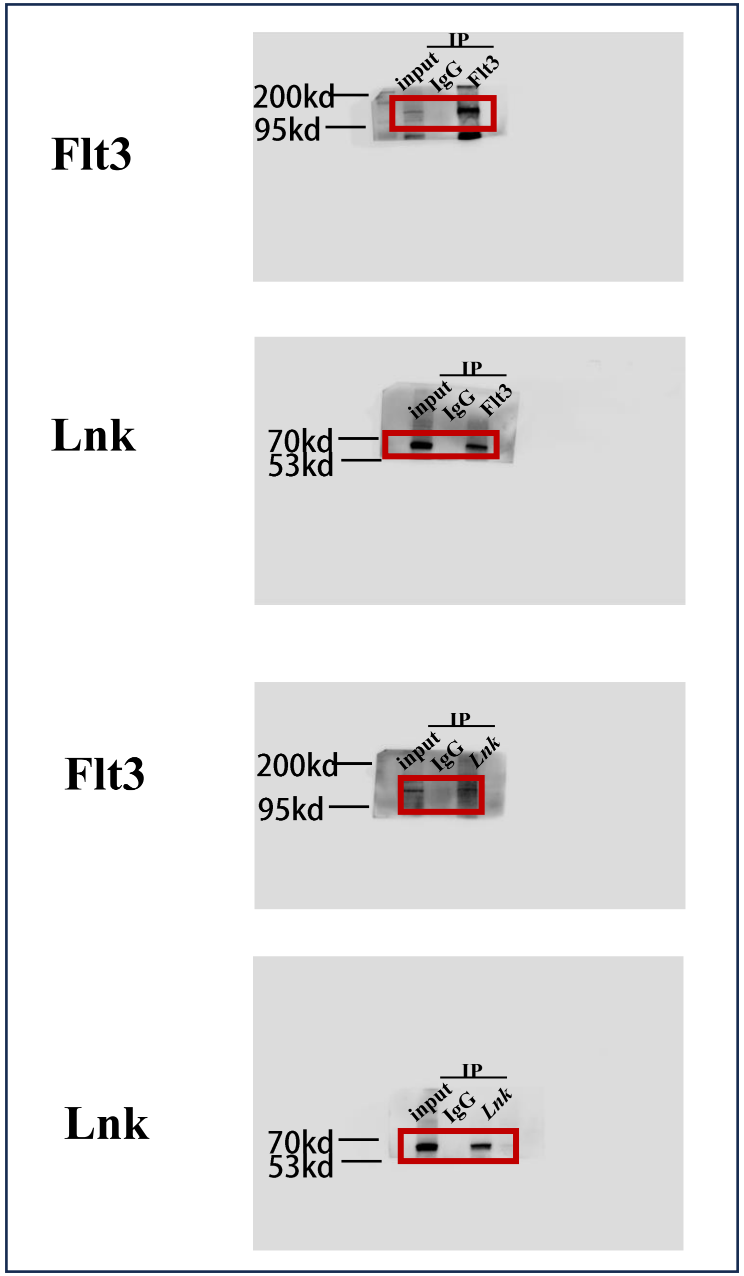

Figure 5F

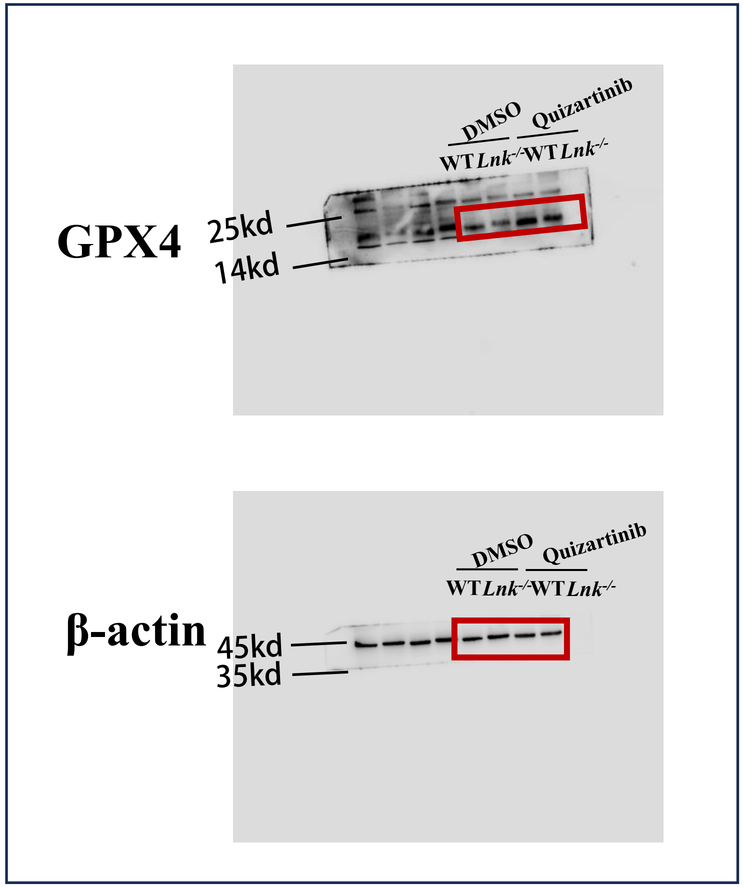

Figure 5I

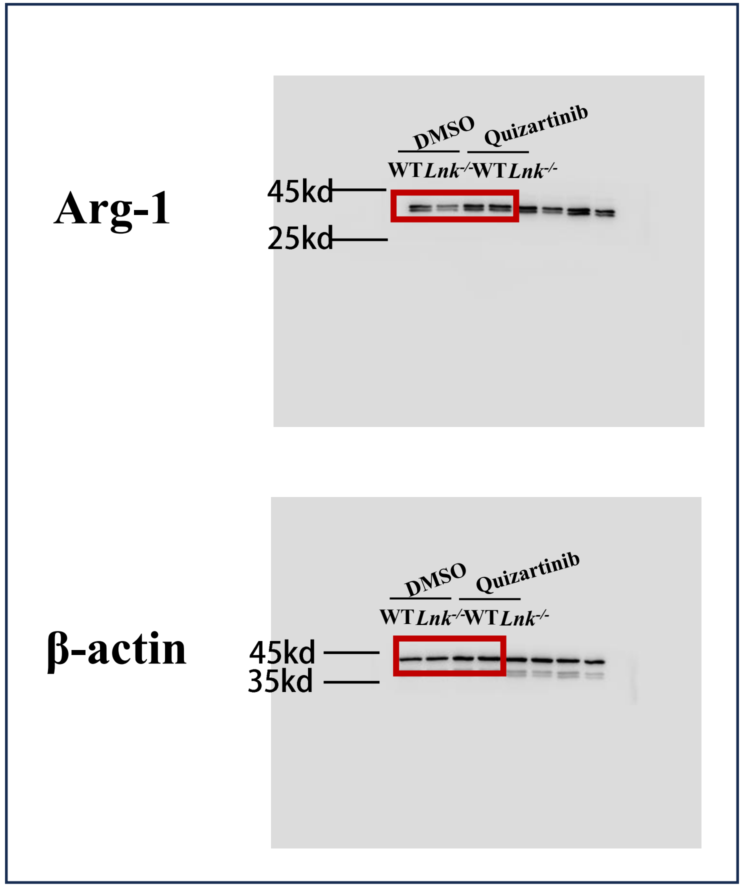

Figure 6C

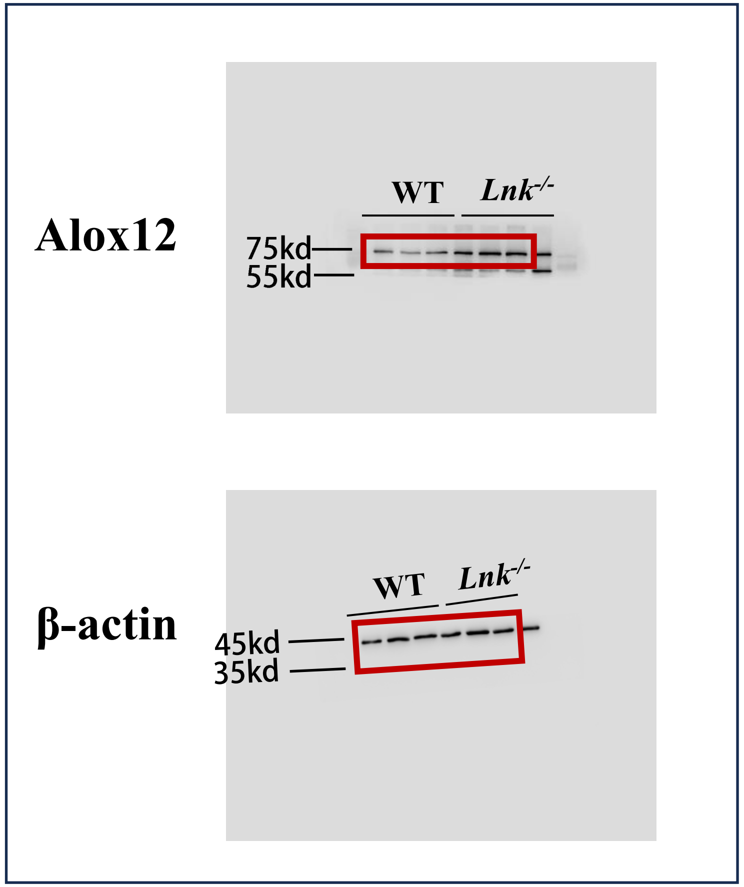

Figure 6D

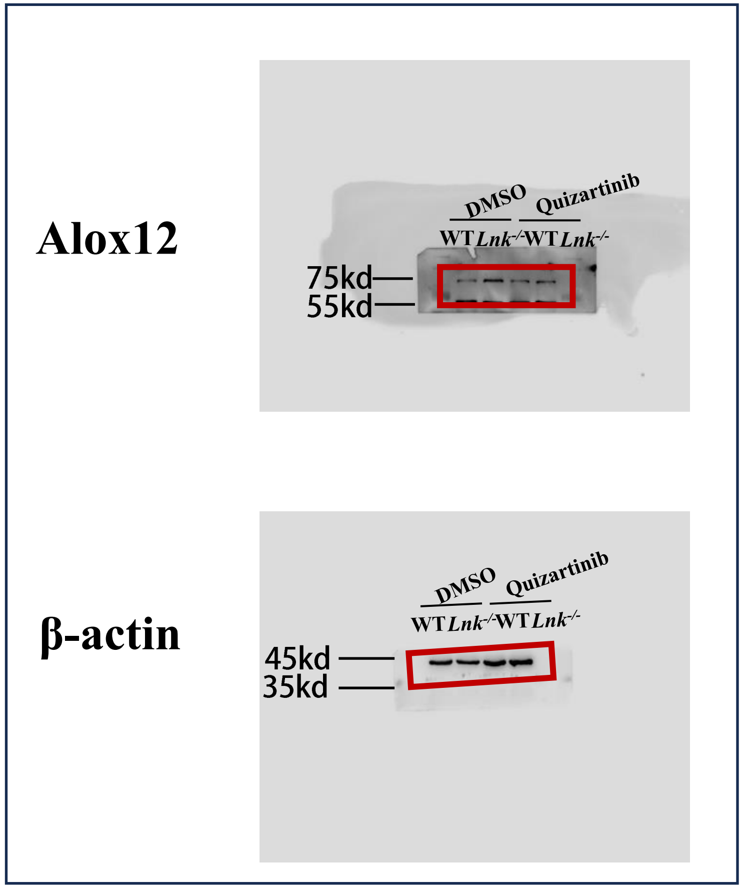

Figure 6E

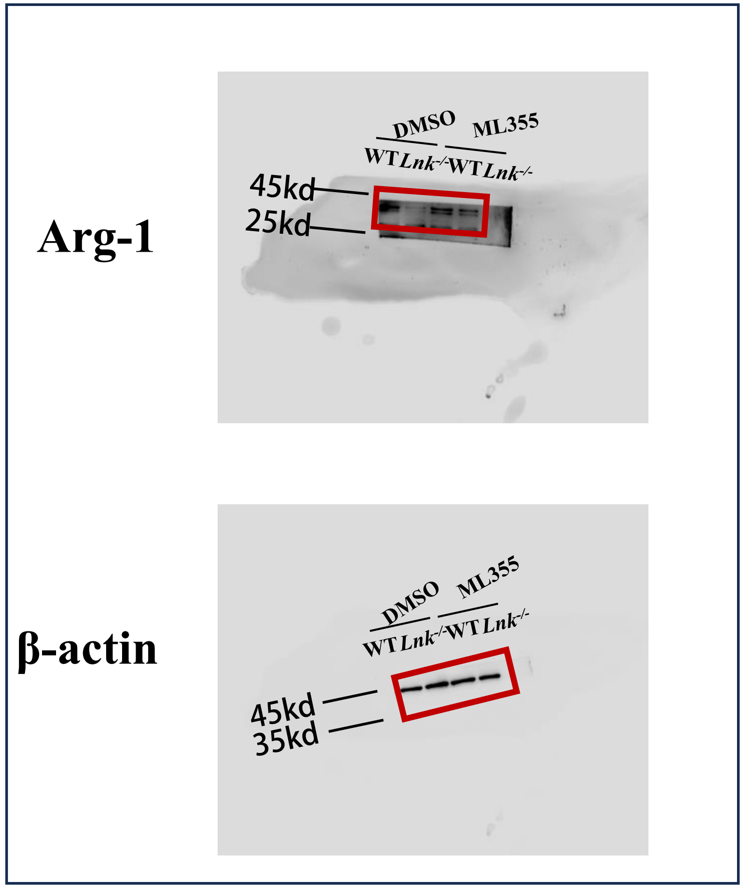

Figure 6G

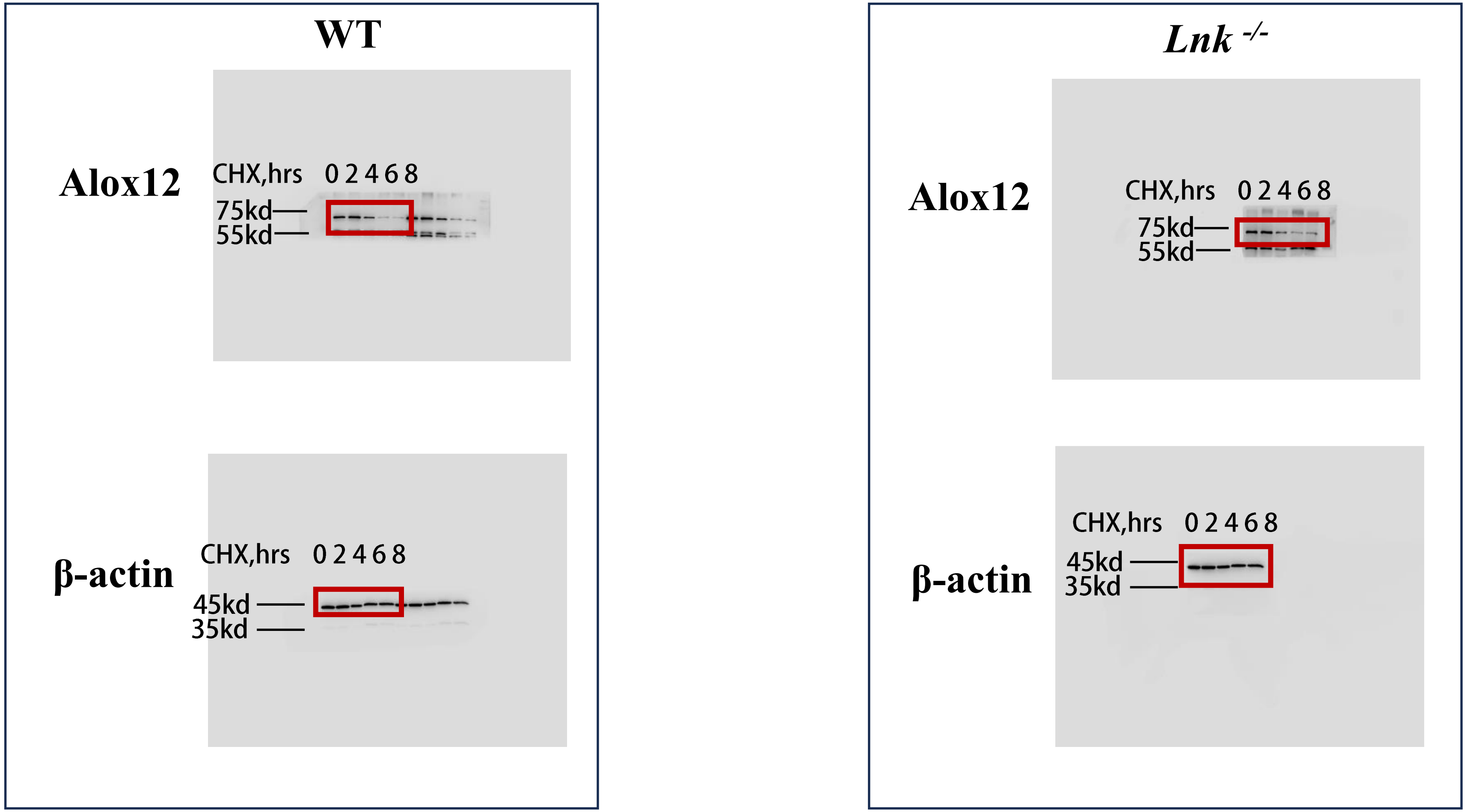
WT
Alox12
CHX,hrs 0 2 4 6 8
75kd
55kd
β-actin
CHX,hrs 0 2 4 6 8
45kd
35kd
Lnk -/-
Alox12
CHX,hrs 0 2 4 6 8
75kd
55kd
CHX,hrs 0 2 4 6 8
β-actin
45kd
35kd

Figure 6I

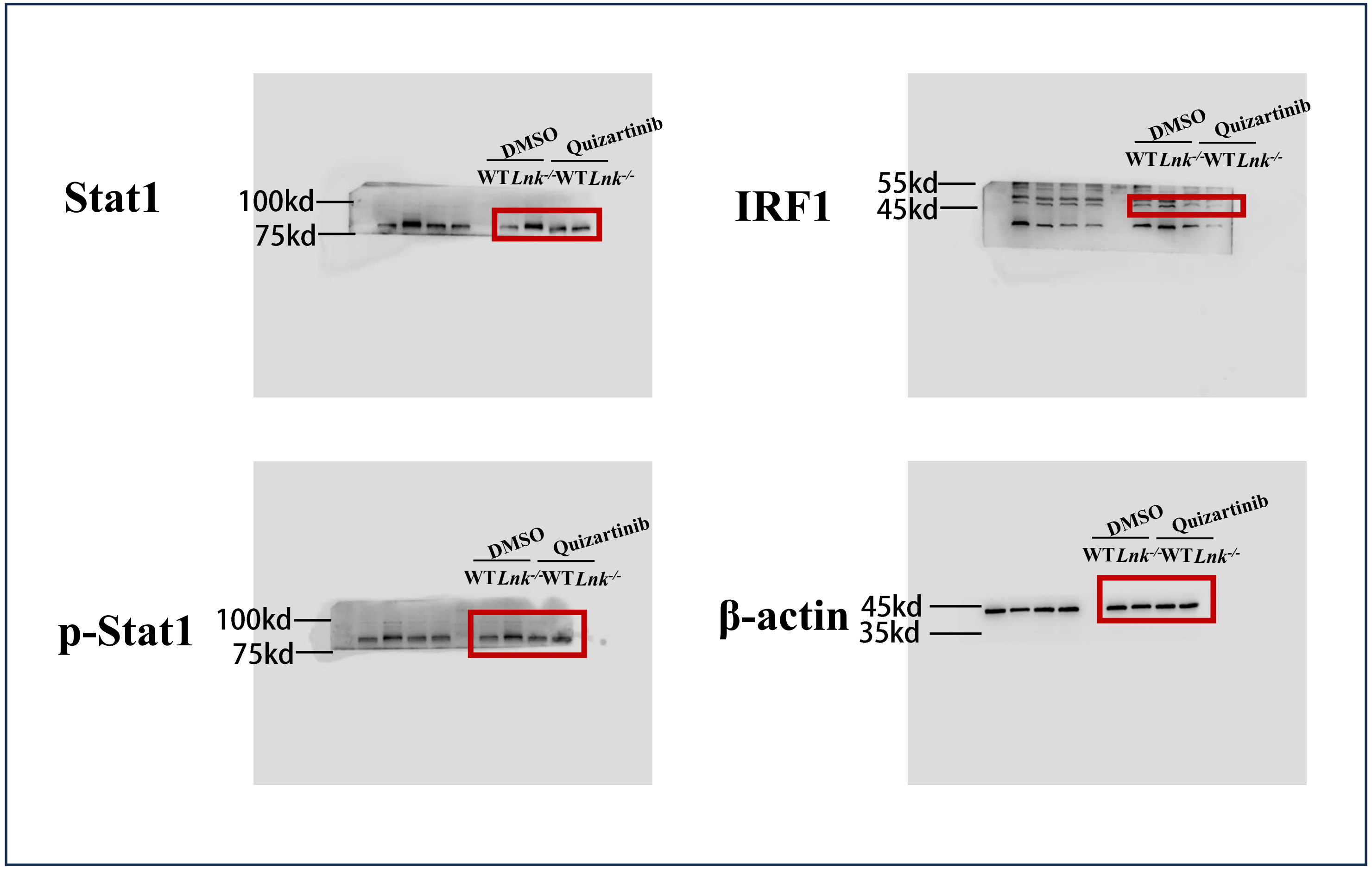

Figure 6J

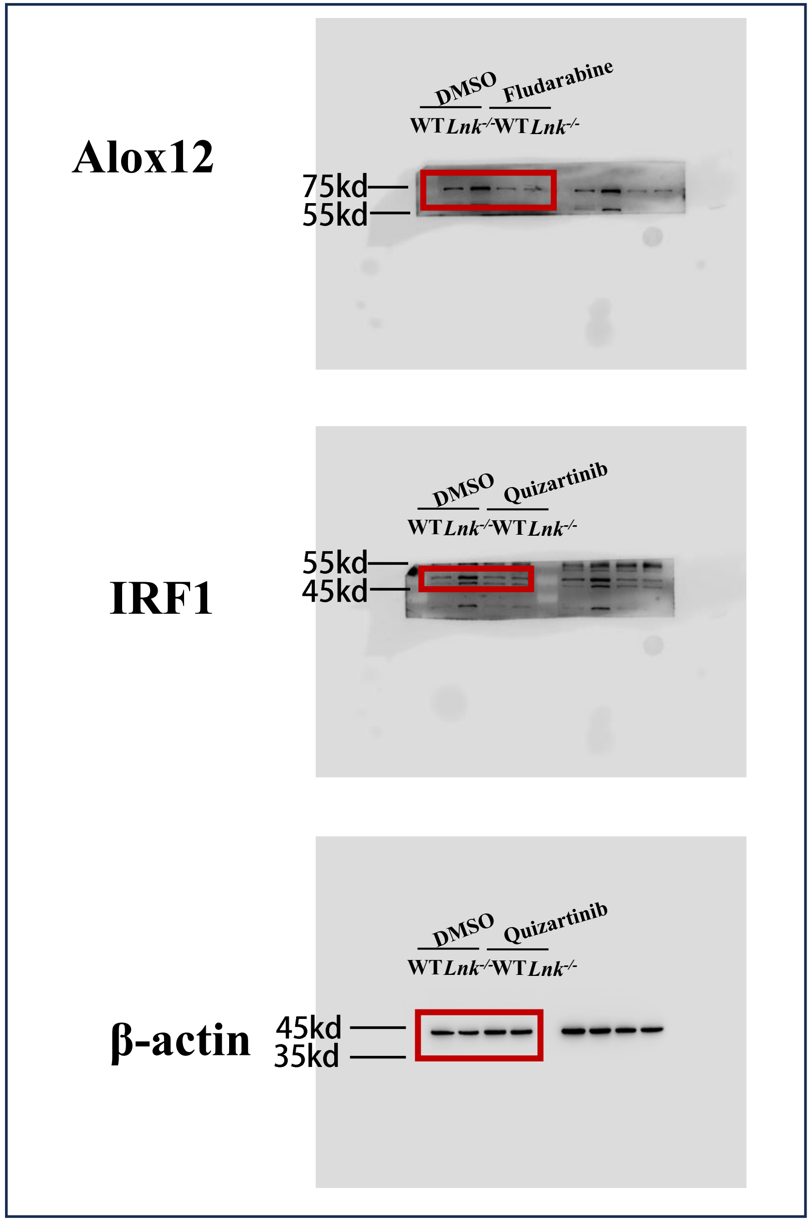

Figure 6K

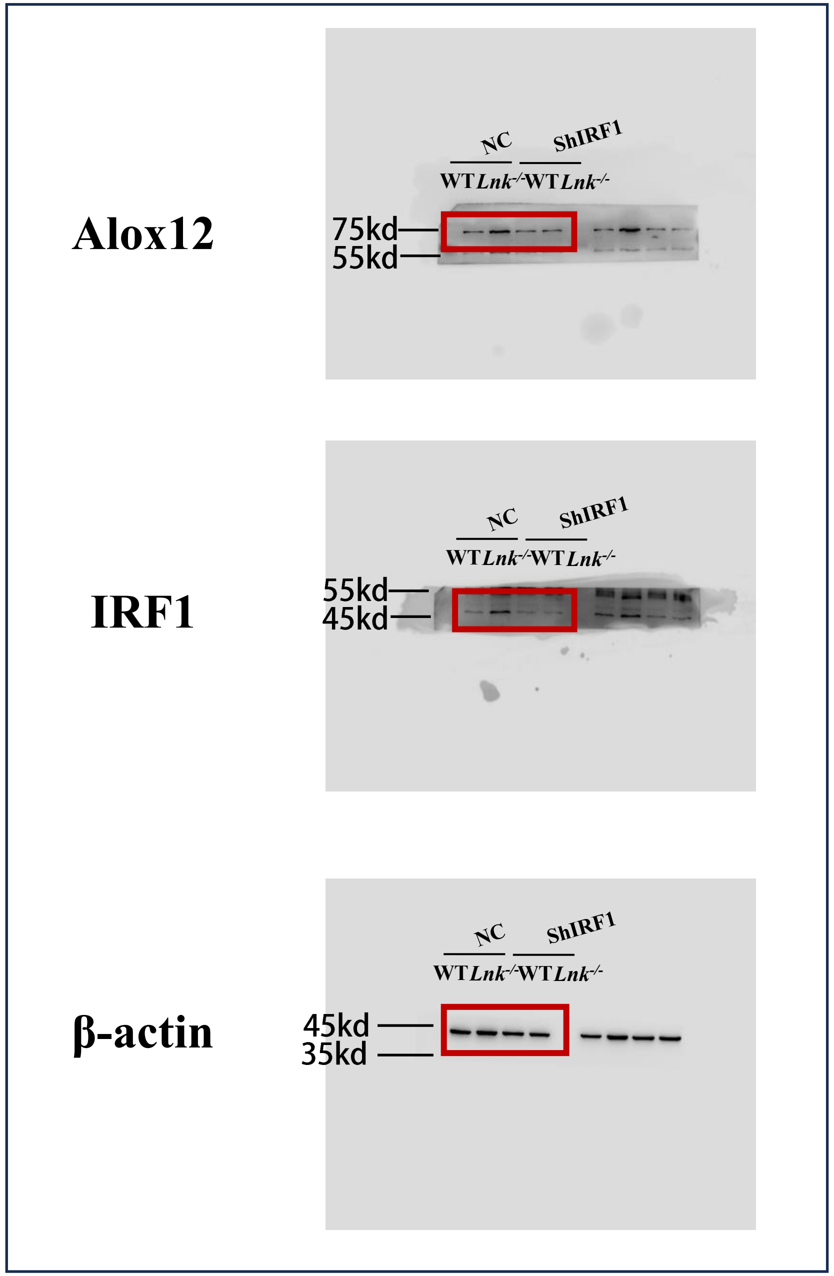
Alox12
NC
ShIRF1
WT Lnk-/- WT Lnk-/-
75kd
55kd
IRF1
NC
ShIRF1
WT Lnk-/- WT Lnk-/-
55kd
45kd
β-actin
NC
ShIRF1
WT Lnk-/- WT Lnk-/-
45kd
35kd

Figure 7C

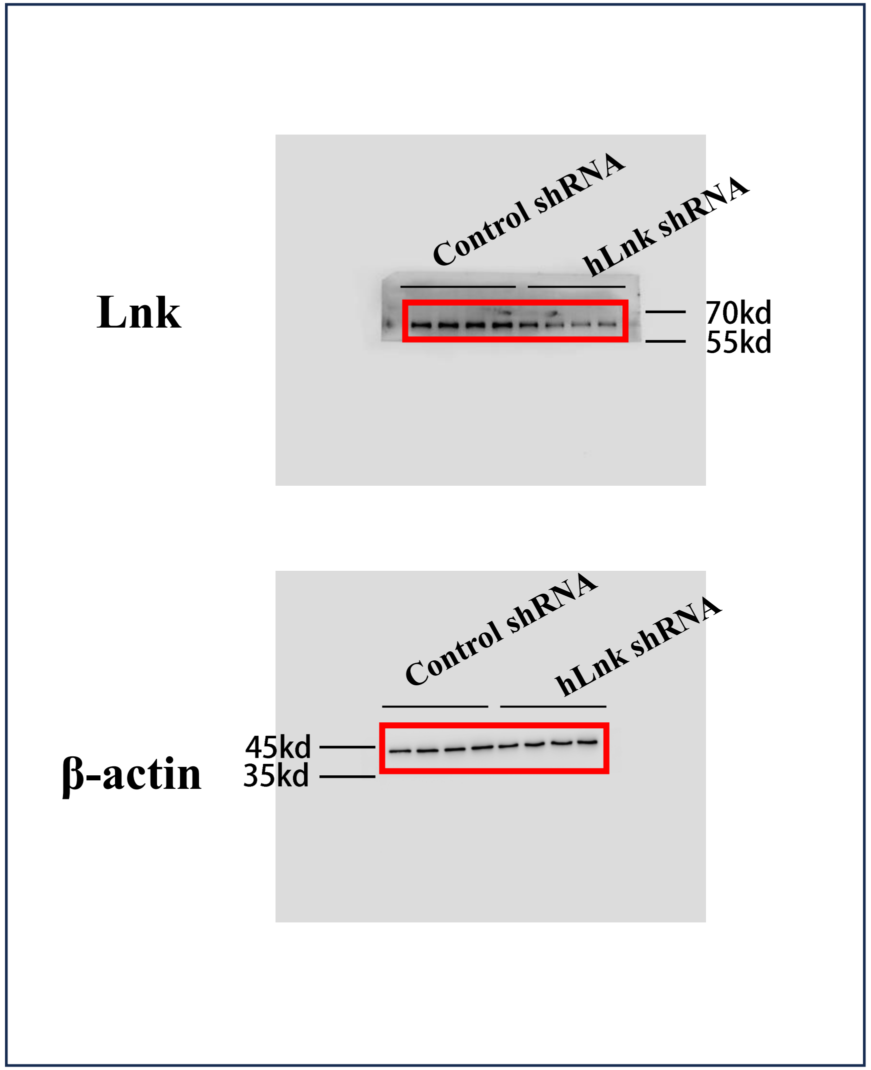

Lnk
Control shRNA
hLnk shRNA
70kd
55kd
β-actin
Control shRNA
hLnk shRNA
45kd
35kd

Supplemental Figure S6

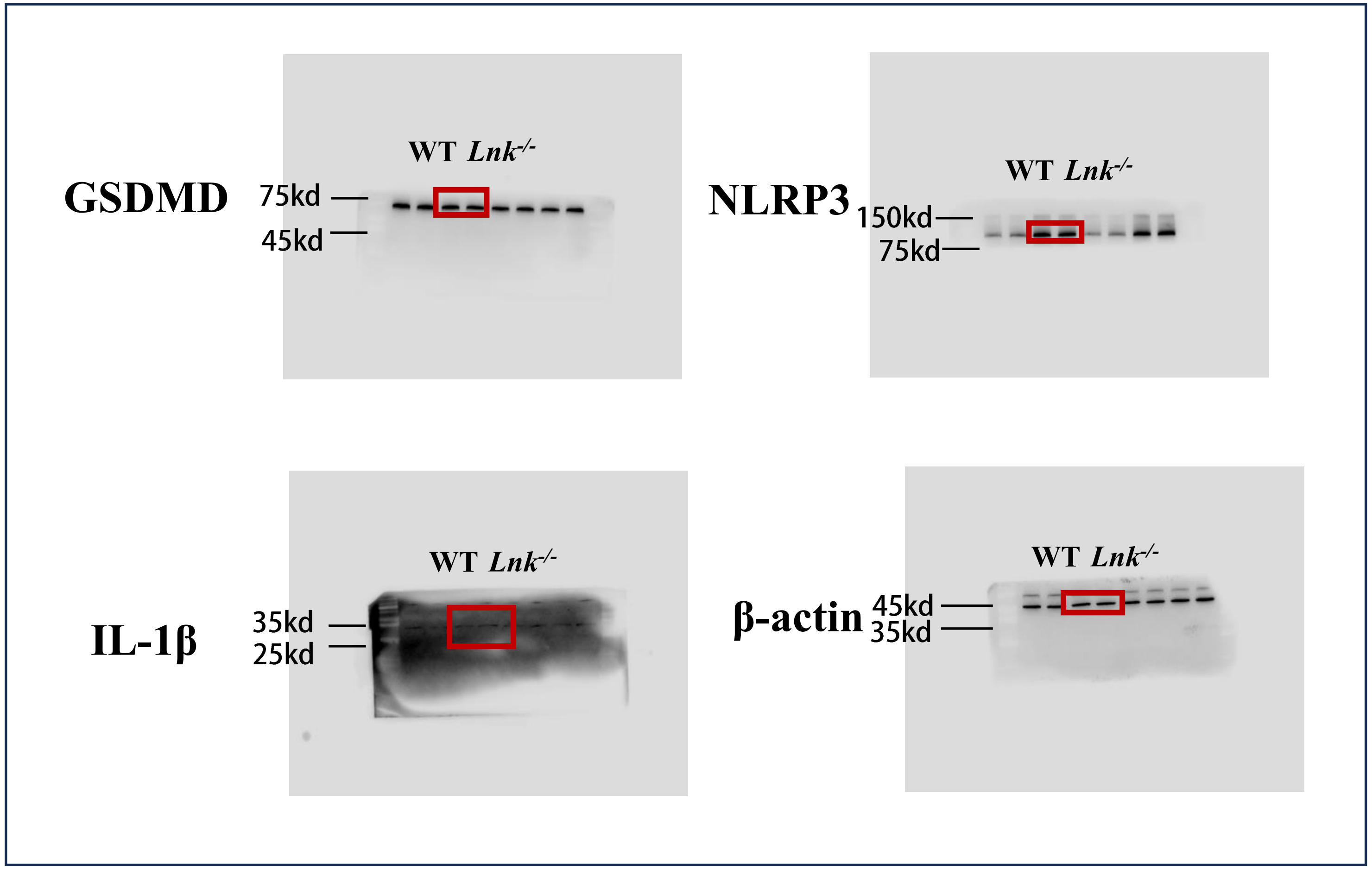

Supplement: Supplementary file 1 — Original western blots. [file 41419_2025_7948_MOESM1_ESM.pdf]
