# Supplementary material for: Lnk deficiency attenuates the immunosuppressive capacity of MDSCs via ferroptosis to suppress tumor development
Source: Cell Death Dis. 2025 Aug 12;16(1):610. doi: 10.1038/s41419-025-07948-8 (PMC12343911; doi:10.1038/s41419-025-07948-8)
Supplement: Supplementary file 3 — Supplemental materials [file 41419_2025_7948_MOESM3_ESM.docx]

**Supplemental Figure S1. The percentages of** **leukocyte subsets, such as IMCs, macrophages, DCs, CD3^+^ T cells, B cells, NK cells and Tregs, in the spleen did not differ between WT and *Lnk^-/-^* mice. (A-B)** Tumor growth kinetics**(A)** and tumor weights**(B)** in WT and *Lnk^-/-^* C57BL/6 mice (n=4 mice/group) bearing B16F10 melanoma cells. **(C)** Representative flow cytometry results and statistical graph showing splenic leukocyte subsets from WT and *Lnk^-/-^* mice. FSC, forward scatter. SSC, side scatter. Data are shown as means ± SEM. *P < 0.05; **P < 0.01; ***P < 0.001; ns, not significant.

**
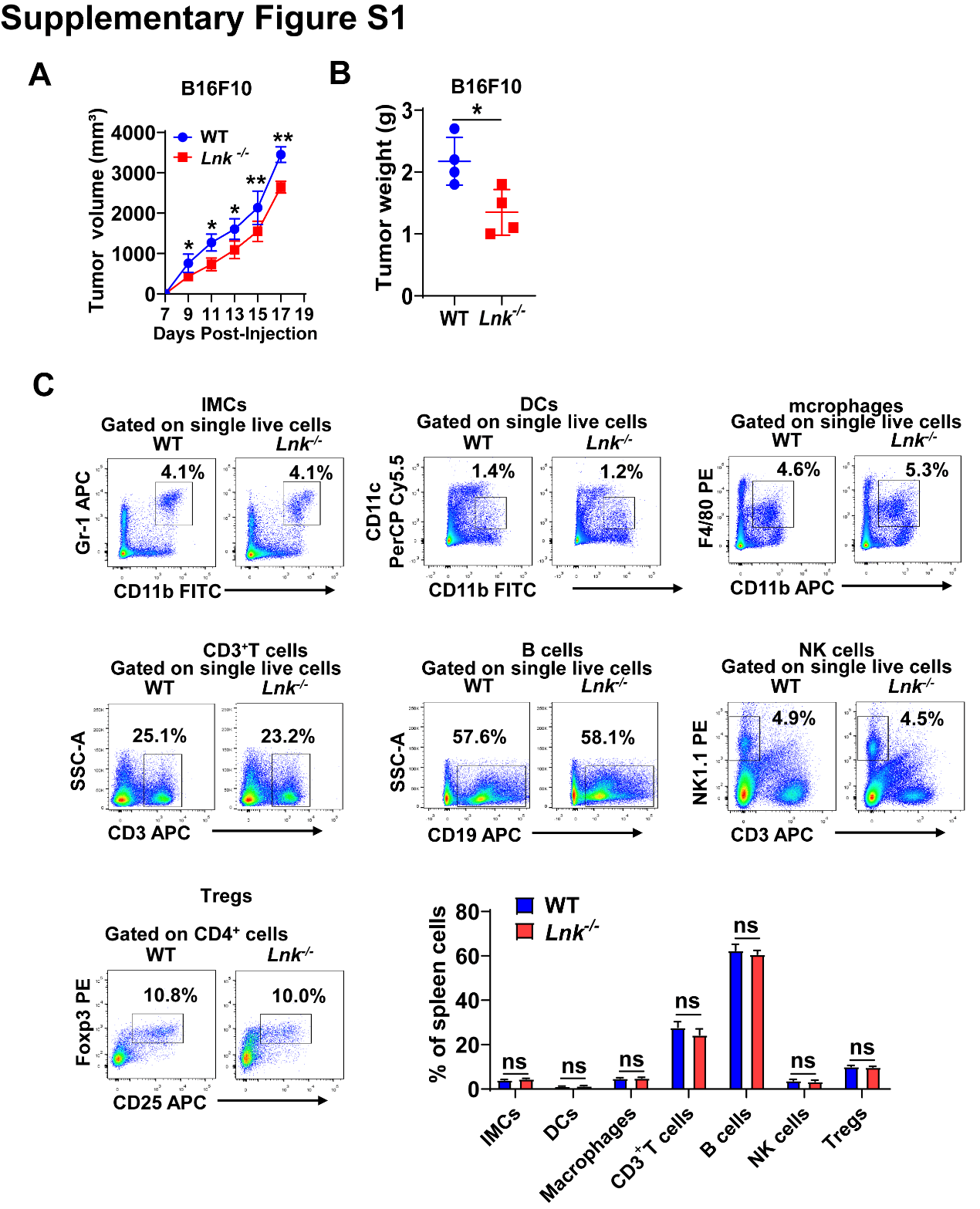
**

**Supplemental Figure S2. Flow cytometry analyses of** **splenic leukocyte subsets in 3LL/B16F10-bearing WT and *Lnk*^-/-^mice.** **(A-B)** Percentages of leukocyte subsets among live cells isolated from the spleens of WT and *Lnk*^-/-^ mice, as determined by flow cytometry after tumor cell injection. MDSCs were CD11b^+^Gr-1^+^ (n=4); G-MDSCs were CD11b^+^Ly6G^+^Ly6C^low^ (n=3); M-MDSCs were CD11b^+^Ly6G^-^Ly6C^high^ (n=3); DCs were CD11b^+^CD11c^+^ (n=4); macrophages were CD11b^+^F4/80^+^ (n=4); CD3^+^ T cells were CD3^+^ (n=4); CD4^+^ T cells were CD3^+^CD4^+^ (n=3); CD8^+^ T cells were CD3^+^CD8^+^ (n=3); B cells were CD19^+^ (n=3); NK cells were CD3^-^NK1.1^+^ (n=3); and Tregs were CD4^+^CD25^+^Foxp3^+^ (n=3). **(A)** Representative and statistical flow cytometry results showing leukocyte subsets from the spleens of WT and *Lnk^-/-^* mice on day 15 after B16F10 cell injection. FSC, forward scatter; SSC, side scatter. **(B)** Representative flow cytometry results showing leukocyte subsets from the spleens of WT and *Lnk^-/-^* mice on day 21 after 3LL cell injection, as shown in Fig. 1C. **(C)** Flow cytometry was performed to assess the numbers of MDSCs and subsets in the B16F10 tumor model (n=3). Data are shown as means ± SEM. *P < 0.05; **P < 0.01; ***P < 0.001; ns, not significant.

**
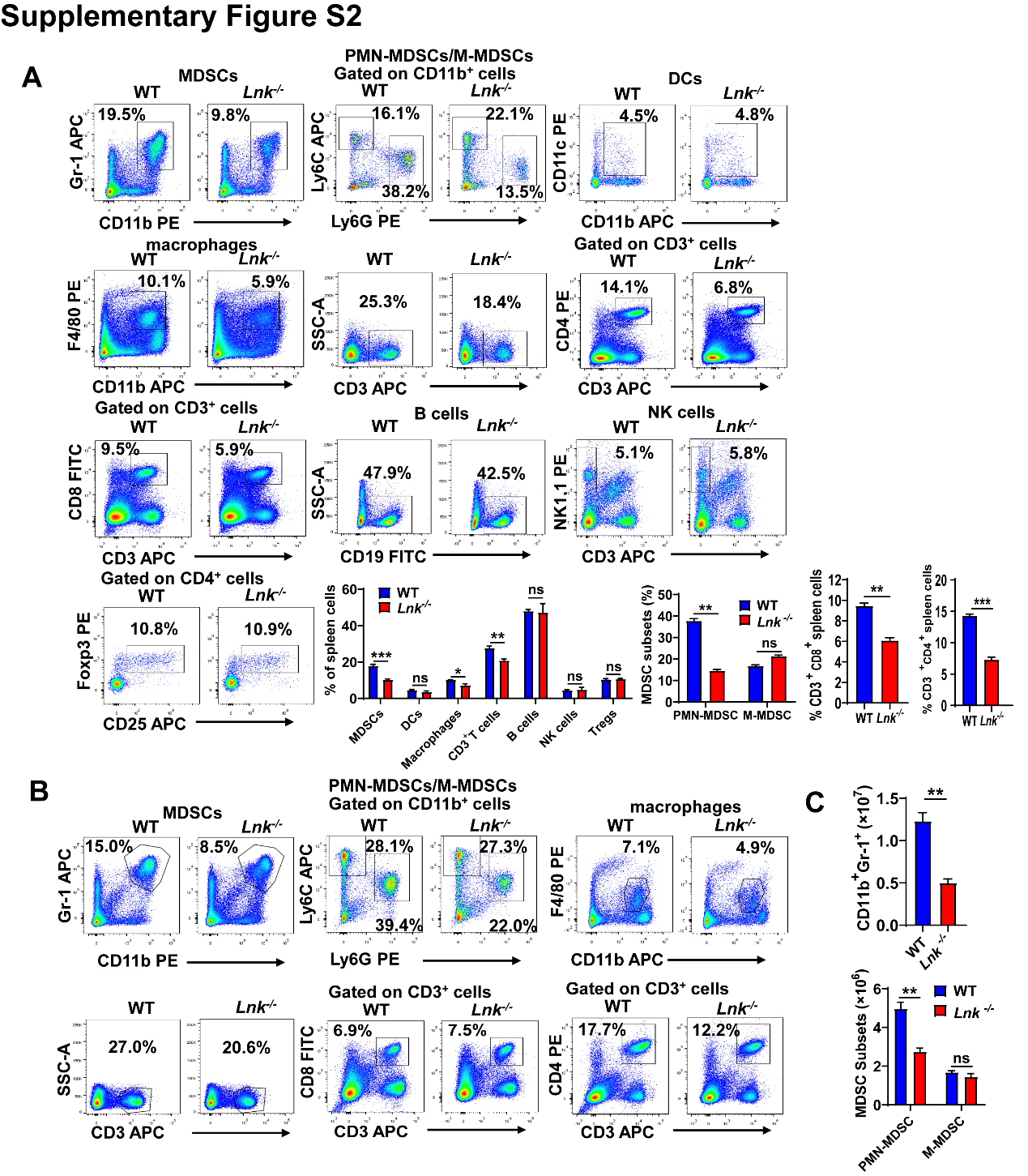
**

**Supplemental Figure S3. Flow cytometry analyses of leukocyte subsets in tumors tissues of WT and *Lnk*^-/-^mice. (A)** The gating strategy diagram of flow cytometry of leukocyte subsets in tumor tissues. **(B-H)** Percentages of leukocyte subsets among live cells isolated from the tumor tissues of WT and *Lnk*^-/-^ 3LL-bearing mice, as determined by flow cytometry after tumor cell injection. **(B)** MDSCs were CD45^+^CD11b^+^Gr-1^+^ (n=4). **(C)** PMN-MDSCs were CD45^+^CD11b^+^Ly6G^+^Ly6C^low^ (n=4); M-MDSCs were CD45^+^CD11b^+^Ly6G^+^Ly6C^high^ (n=4). **(D)** Macrophages were CD45^+^CD11b^+^F4/80^+^ (n=4). **(E)** CD3^+^ T cells were CD45^+^CD3^+^ (n=4). **(F)** CD4^+^ T cells were CD45^+^CD3^+^CD4^+^ (n=3). **(G)** CD8^+^ T cells were CD45^+^CD3^+^CD8^+^ (n=3). **(H)** DCs were CD45^+^CD11b^+^CD11c^+^ (n=4); CD19^+^ B cells were CD45^+^CD3^-^CD19^+^ (n=4); NK cells were CD45^+^CD3^-^NK1.1^+^ (n=4); and Tregs were CD45^+^CD4^+^CD25^+^Foxp3^+^ (n=4). Data are shown as means ± SEM. ns, not significant.

**
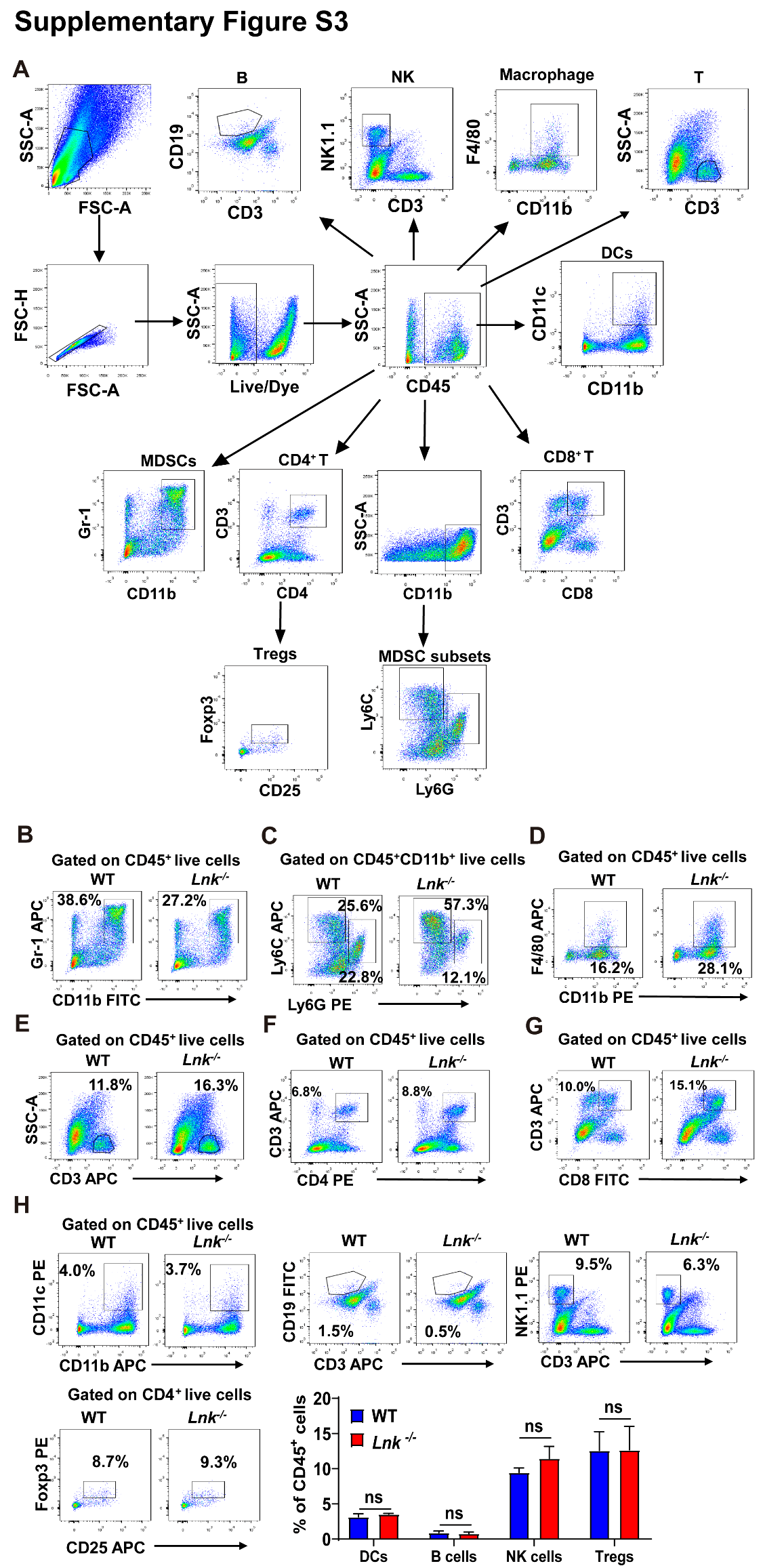
**

**Supplemental Figure S4. Compared with WT MDSCs, *Lnk^-/-^* MDSCs had no significant effect on ROS or PD-L1 expression or CD4^+^ T/CD8^+^ T cell proliferation. The immune-inhibitory activity of Lnk-deficient BM-derived MDSCs decreased in vitro (A)** ROS levels in the spleens of WT and *Lnk^-/-^* mice on day 21 after 3LL cell injection determined by flow cytometry using the ROS fluorescent probe DHE. **(B)** PD-L1 expression in MDSCs (n=3) from the spleens of WT and *Lnk^-/-^* mice was determined by flow cytometry 21 days after the s.c. injection of 3LL cells. **(C-D)** Splenic MDSCs from 3LL tumor-bearing WT and *Lnk^-/-^* mice were cocultured with anti-CD3/CD28-activated CD4^+^ T/CD8^+^ T cells at a ratio of 1:2. Flow cytometry analysis of the proliferation of CD4^+^ T/CD8^+^ T cells was performed via Ki-67 intracellular staining after day 2 (n=3). **(E-I)** BM cells isolated from WT and *Lnk^-/-^* mice were treated with GM-CSF for 48 h. **(E-H)** NOS expression (n=4) **(E)**, Arg-1 activity (n=3) **(F)**, TNF-α secretion (n=4) **(G)** and IFN-γ expression (n=4) **(H)** in MDSCs were measured by a nitric oxide synthase assay kit, colorimetric assay, ELISA and flow cytometry, respectively. **(I)** Percentage of IFN-γ-expressing CD8^+^ T cells (n=6) in cocultures of MDSCs and anti-CD3/CD28-activated CD8^+^ T cells at a ratio of 1:2, as determined by flow cytometry. Data are shown as means ± SEM. *P < 0.05; **P < 0.01; ***P < 0.001; ns, not significant.


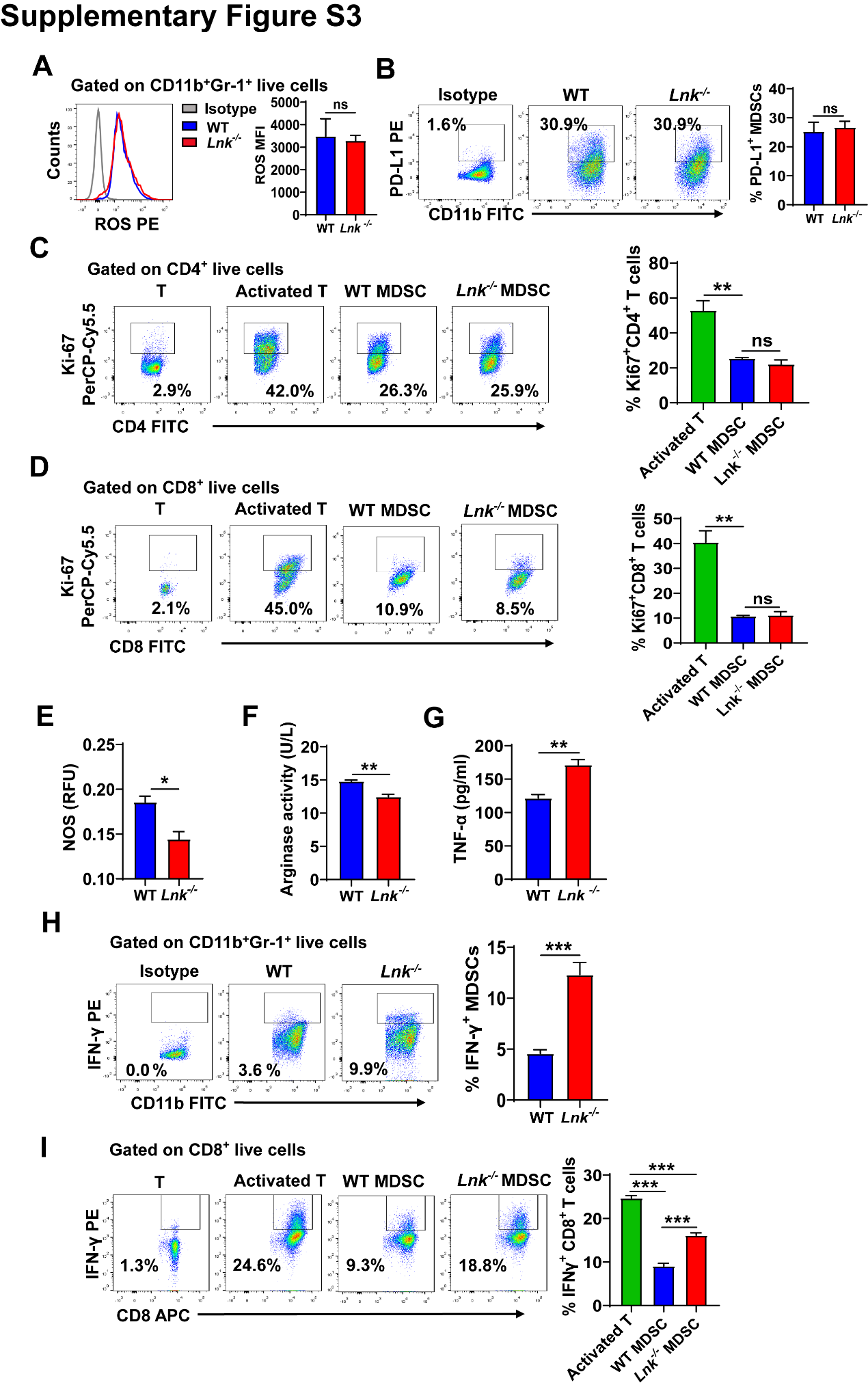


**Supplemental Figure S5. Lnk deficiency weakened the immunosuppressive function of tumor MDSCs. (A and G)** The expression of MHC-II **(A)**, MHC-I **(B)**, TNF-α **(C),** IFN-γ **(D)** and Arg-1 **(E)** in MDSCs (n=4) from the tumor tissues of WT and *Lnk^-/-^*C57BL/6 mice that were s.c. injected with 3LL cells was determined by flow cytometry 21 days after injection. **(F)** Representative images of immunofluorescence double staining of Gr-1 (red) and iNOS (green). The cell nuclei were stained with DAPI (blue). **(G and H)** The percentage of Ki67^+^CD8^+^T and IFN-γ^+^CD8^+^T in tumor tissues of WT and *Lnk^-/-^* 3LL bearing-mice were analyzed with flow cytometry, n=4. **(I and J)** Percentage of IFN-γ-expressing and Ki67-expressing CD8^+^ T cells (n=4) in cocultures of tumor MDSCs and CD8^+^ T cells at a ratio of 1:2, as determined by flow cytometry. MDSCs were isolated from the tumor tissues of WT and *Lnk^-/-^* mice 21 days after s.c. injection of 3LL cells. CD8^+^ T cells isolated from the spleens of C57BL/6 mice were activated in vitro with anti-CD3 and anti-CD28 antibodies for 48 h before coculture; unactivated T cells were used as the control. **(K)** Representative flow cytometry results of Fig3 J. Data are shown as means ± SEM. *P < 0.05; **P < 0.01; ***P < 0.001; ns, not significant.


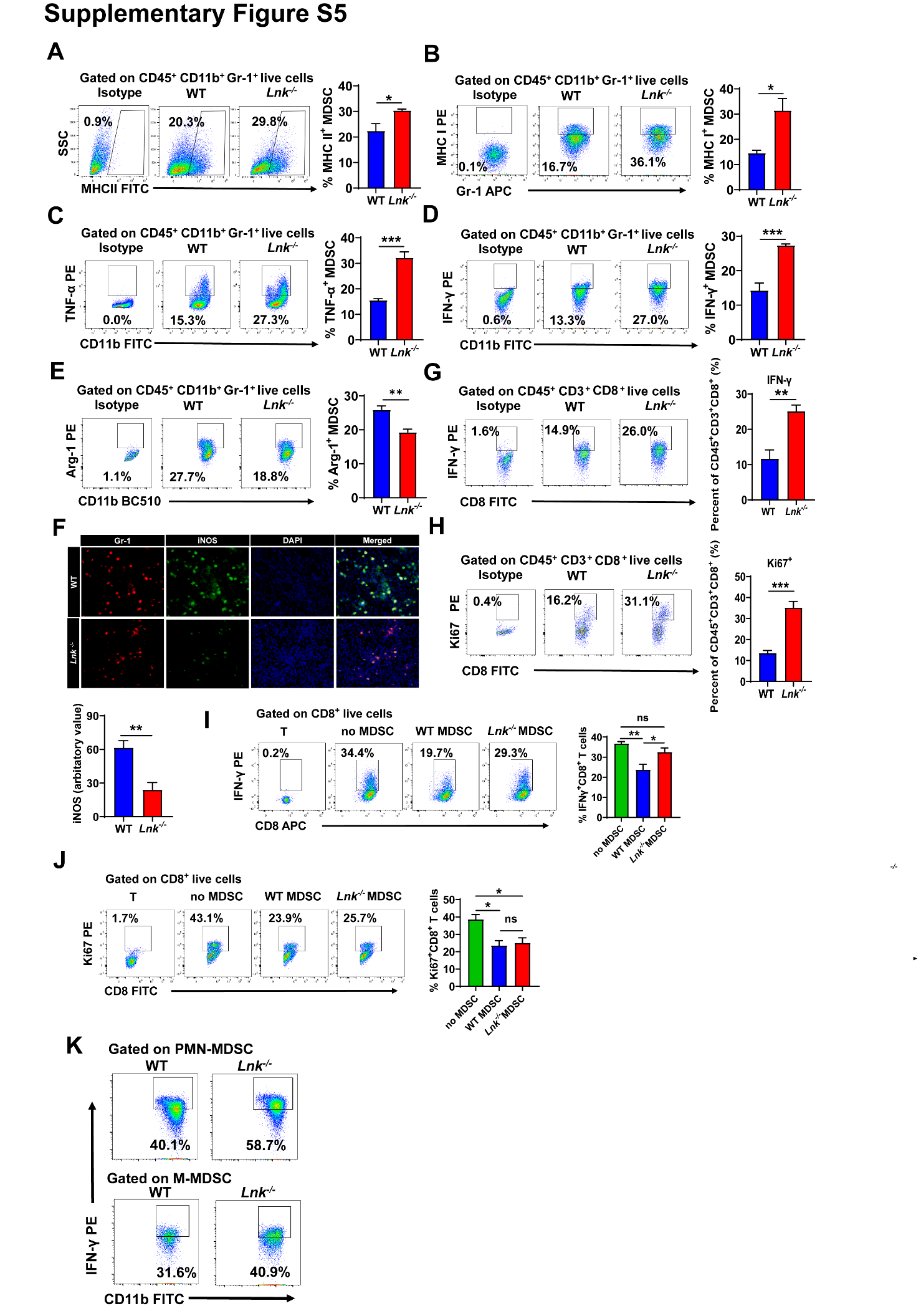


**Supplemental Figure S6. Compared with WT MDSCs, *Lnk^-/-^* MDSCs had no significant effect on** **cell proliferation, apoptosis and** **pyroptosis. (A)** The percentage of proliferating (Ki67^+^) MDSCs in spleens of WT and *Lnk^-/-^* tumor-bearing mice were analyzed with flow cytometry, n=4. **(B)** Representative staining and frequencies of AnnexinV^+^ PI^+^ cells in MDSCs from spleens of WT and *Lnk^-/-^* tumor-bearing mice were assessed by flow cytometry, n=4. **(C)** Western blot analysis of NLRP3, GSDMD and IL-1β in MDSCs isolated from the spleens of 3LL-bearing WT and *Lnk^-/-^* mice, n=4. Data are shown as means ± SEM. *P < 0.05; **P < 0.01; ***P < 0.001; ns, not significant.

**
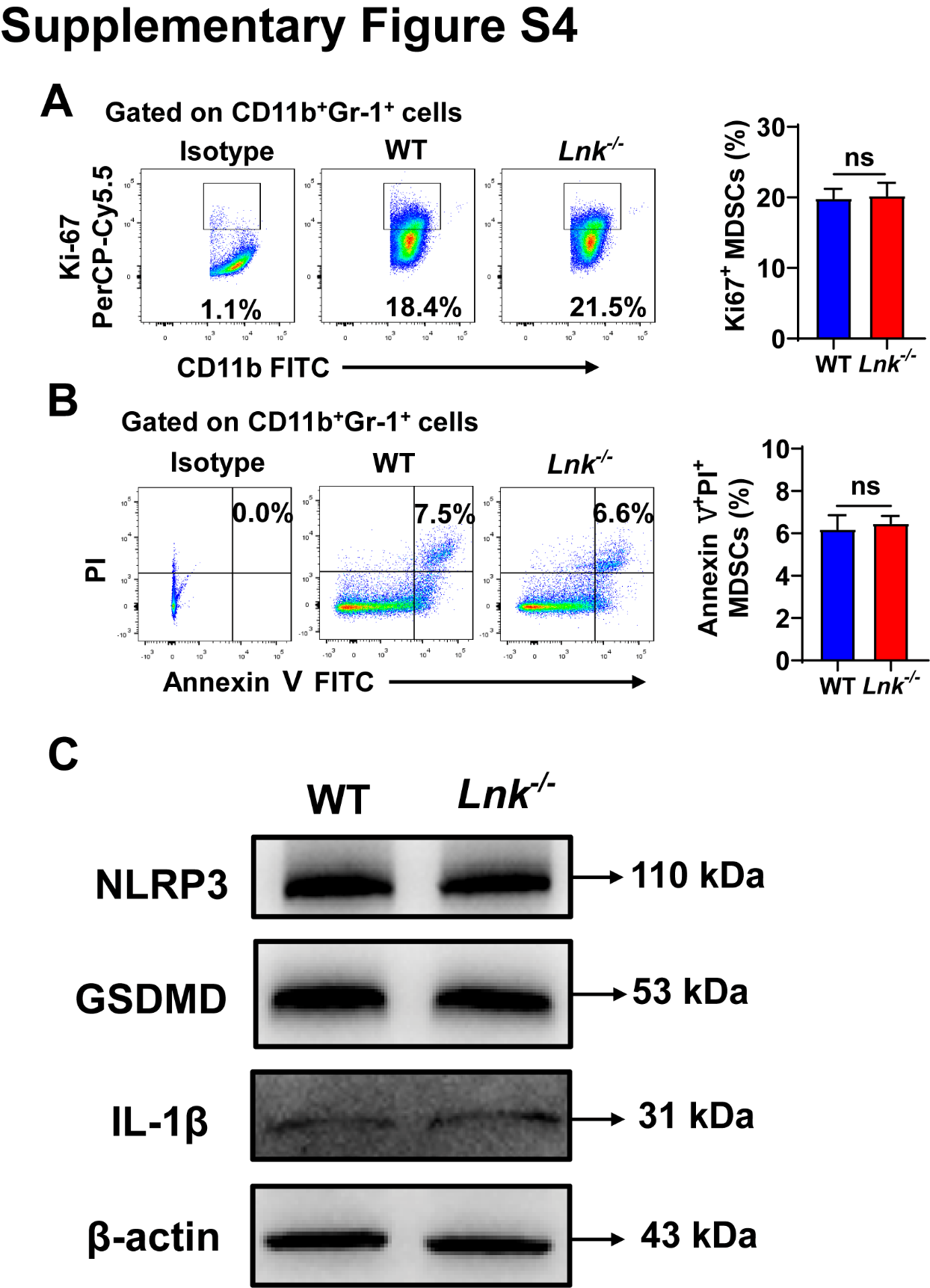
**

**Supplemental Figure S7. IKE treatment inhibited tumor growth and reduced the immunosuppressive function of MDSCs in *Lnk^-/-^* mice. (A)** A schematic representation of the experimental design; WT and *Lnk^-/-^* mice were injected s.c. with 3LL cells on day 0. Then, IKE (10mg/kg/day) was injected i.p. at day 9 till day 21, DMSO treatment was used as the control group. **(B and C)** Tumor growth curve **(B)** and tumor weight **(C)** in WT and *Lnk^-/-^* mice treated without/with IKE, n = 4 mice/group. **(D)** Targeted phospholipid analysis using the HPLC-MS/MS of ferroptotic cell death signals (PE-16:0/22:4-OOH) and (PE-18:0-20:5-OOH) of MDSCs in tumors from WT and *Lnk^-/-^* mice treated without/with IKE, n = 4. **(E and F)** The expression of TNF-α **(E)** and Arg-1 **(F)** in MDSCs from the tumor tissues of WT and *Lnk^-/-^* mice treated without/with IKE were determined by flow cytometry, n = 4. **(G)** Percentage of IFN-γ-expressing CD8^+^ T cells (n=4) in cocultures of tumor MDSCs and CD8^+^ T cells at a ratio of 1:2, as determined by flow cytometry. Tumor MDSCs were isolated from the tumor tissues of WT and *Lnk^-/-^* 3LL-bearing mice treated without/with IKE. CD8^+^ T cells isolated from the spleens of C57BL/6 mice were activated in vitro with anti-CD3 and anti-CD28 antibodies for 48 h before coculture; unactivated T cells were used as the control. Data are shown as means ± SEM. *P < 0.05; **P < 0.01; ***P < 0.001; ns, not significant.

**
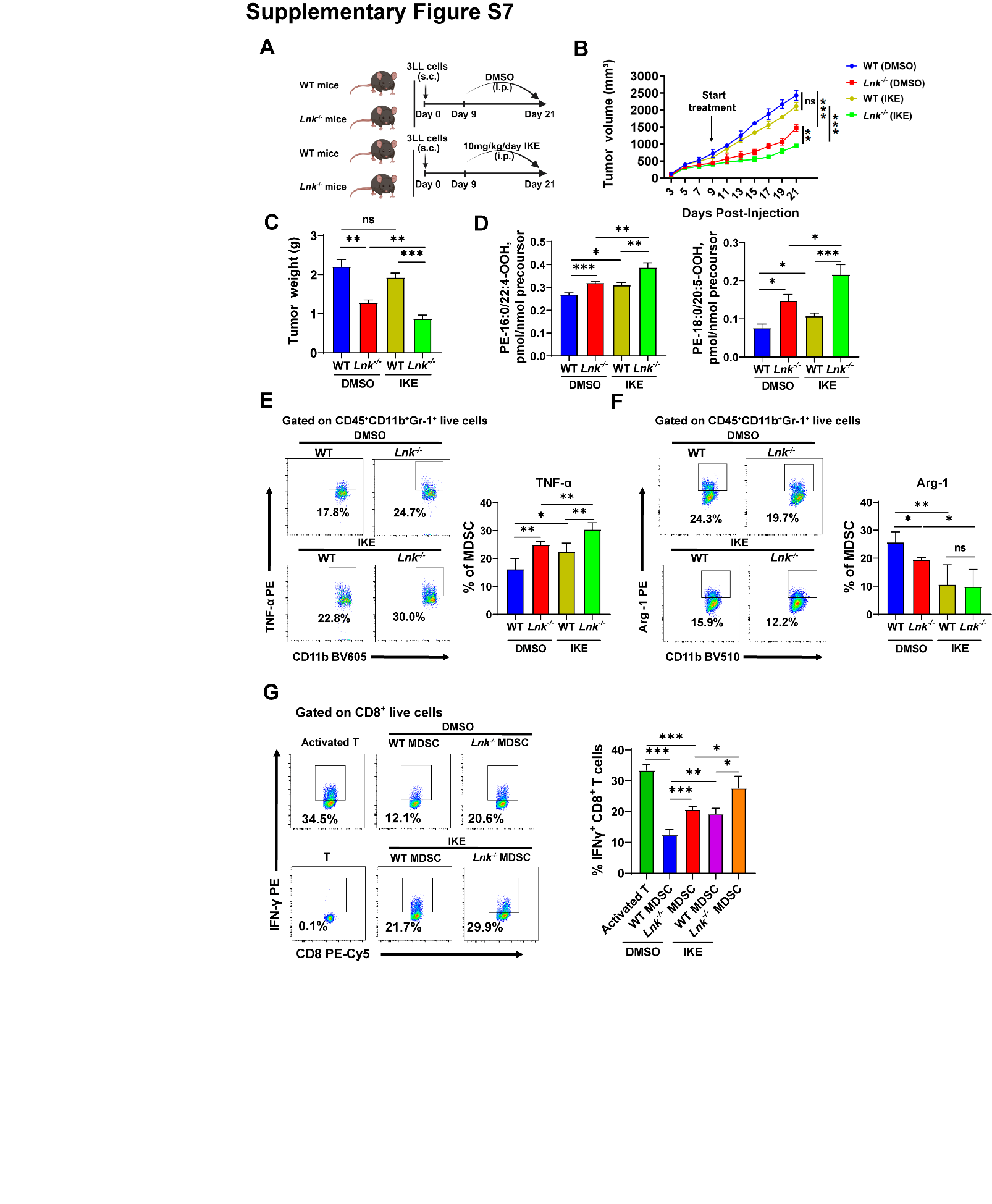
**
